# Integrated immunoinformatics for the design of novel multi-epitope vaccine and identification of new drug targets against *Stenotrophomonas maltophilia,* a multidrug-resistant superbug

**DOI:** 10.1016/j.jgeb.2026.100772

**Published:** 2026-07-28

**Authors:** Safoura Moradkasani, Narjes Noori Goodarzi, Masoumeh Beig, Mohammad Sholeh, Sepideh Fereshteh, Mahshid Khazani Asforooshani, Behzad Shahbazi, Shahla Shahbazi, Farzad Badmasti

**Affiliations:** a Department of Bacteriology, Pasteur Institute of Iran, Tehran, Iran; b Department of Epidemiology and Biostatics, Research Centre for Emerging and Reemerging Infectious Diseases, Pasteur Institute of Iran, Tehran, Iran.; c Department of Pathobiology, School of Public Health, Tehran University of Medical Sciences, Tehran, Iran.; d Department of Microbiology, Faculty of Biological Sciences, Alzahra University, Tehran, Iran; e School of Pharmacy, Semnan University of Medical Sciences, Semnan, Iran.; f Nervous System Stem Cells Research Center, Semnan University of Medical Sciences, Semnan, Iran.; g Infectious Diseases Research Center, Health Institute, Kermanshah University of Medical Sciences, Kermanshah, Iran

**Keywords:** *Stenotrophomonas maltophilia*, Reverse vaccinology, Putative immunogenic targets, Multi-epitope vaccine, Drug targets

## Abstract

**Background:**

*Stenotrophomonas maltophilia* is a multidrug-resistant opportunistic pathogen causing severe hospital-acquired infections, especially in immunocompromised patients. The absence of an effective vaccine and rising antibiotic resistance underscore the need for novel interventions. This study employed an integrated reverse vaccinology and computational analyses to identify new immunogenic targets, design a multi-epitope vaccine (MEV), and propose potential drug targets*.*

**Methods:**

A comprehensive immunoinformatics pipeline was employed to assess antigenicity, allergenicity, human similarity, and physicochemical properties of *S. maltophilia* proteins. Both B- and T-cell epitopes were screened; however, only the top B-cell epitopes were selected for MEV construction, given the extracellular nature of *S. maltophilia*. MEV–TLR interactions were analyzed through molecular docking and dynamics simulations. In parallel, cytoplasmic proteins were screened *via* a subtractive genomics approach to identify essential, non-human homologous, and non-microbiome-similar proteins, which were further evaluated for druggability and interaction networks to propose novel therapeutic targets.

**Results:**

From a total of 4111 proteins, seven potential immunogenic targets were identified: GspD (WP_108270537.1), FhuE (WP_049451370.1), fimbrial protein (WP_012479122.1), TonB-dependent receptor (WP_169448402.1), TolC family protein (WP_108270106.1), autotransporter beta-barrel OMP (WP_169448945.1), and a hypothetical protein (WP_005407892.1). Subsequently, an MEV was designed using five immunogenic epitopes derived from four of these targets: WP_005407892.1 (ADQDSSNM), WP_049451370.1 (SGKAEQ and GEESKTPS), WP_108270537.1 (GVTSTQSDSERT), and WP_169448945.1 (RELGGDRNE). Molecular docking and molecular dynamics simulations demonstrated strong, stable, and feasible interactions between the MEV and TLR-2 and TLR-4 receptors. Moreover, nine novel drug targets were predicted for *S. maltophilia*, providing new therapeutic insights.

**Conclusion:**

The designed MEV and identified immunogenic targets represent promising vaccine candidates against *S. maltophilia*. Further *in vitro* and *in vivo* studies are essential to confirm their safety, immunogenicity, and protective efficacy. Additionally, subtractive genomics analysis revealed nine novel, non-homologous drug targets, offering safer and more specific therapeutic avenues.

## 1 Introduction

*Stenotrophomonas maltophilia* is an opportunistic, ubiquitous pathogen. It ranks as the third most frequently encountered non-fermenting Bacillus species in healthcare settings, trailing *Pseudomonas aeruginosa* and Acinetobacter species ^1^. Immunosuppression, malignancy, indwelling medical devices, mechanical ventilation, and broad-spectrum antibiotic therapy are the main risk factors associated with *S. maltophilia* infections.^2^ Respiratory tract infections, particularly cystic fibrosis, are the most common clinical manifestations of *S. maltophilia* infection. *S. maltophilia* demonstrates considerable virulence during bacteremia, with mortality rates reported to be between 21% and 69% among those affected. The presence of *S. maltophilia* further complicates the clinical scenario, with polymicrobial infections demonstrating a high mortality rate of 45.8%. *S. maltophilia* colonization and infection are increasingly prevalent in ICU settings and have been classified as a hazardous, drug-resistant pathogen commonly found in healthcare settings.^3^

The emergence and dissemination of multidrug-resistant *S. maltophilia* strains have led to an increase in both nosocomial and community-acquired infections, resulting in elevated morbidity and mortality.^4^ This pathogen poses a significant public health threat, particularly to immunocompromised individuals, with a mortality rate exceeding 37.5%. Its contribution to healthcare-associated infections is compounded by the diminishing susceptibility to commonly used antibiotics, including trimethoprim-sulfamethoxazole, doxycycline, minocycline, respiratory quinolones, and ceftazidime, making the management of *S. maltophilia* infections increasingly challenging.^5^ The growing burden of multidrug-resistant *S. maltophilia* underscores the urgent need for novel therapeutic strategies, including prophylactic vaccines, and highlights the importance of identifying new drug targets.^6^, ^7^ Key virulence factors, such as the Type IV Secretion System (T4SS), have emerged as attractive vaccine targets because of their pivotal role in pathogenicity.^8^ In addition, several immunogenic outer membrane proteins (Omps), including OmpA and Smlt4123, have been characterized, with recombinant Smlt4123 demonstrating protective efficacy in murine models.^9^ These findings emphasize the potential of protein-based vaccines that exploit specific bacterial antigens to induce protective immunity. Due to their abundance and strong immunogenicity, Omps remain central to vaccine research, as they are readily recognized by the host immune system and elicit robust defensive responses.^9^ Traditional vaccine development is often limited by long production times and high expenses. Reverse vaccinology, which leverages advances in sequencing technologies, provides a faster and more cost-effective alternative, as exemplified by the successful development of the meningococcal B vaccine Bexsero®.^10^, ^11^, ^12^, ^13^ This approach offers a systematic framework for identifying and prioritizing vaccine candidates for diverse pathogens. In particular, multi-epitope vaccines (MEVs) have been successfully designed using reverse vaccinology pipelines against pathogens such as Candida auris, human cytomegalovirus, SARS-CoV-2, *Mycobacterium tuberculosis*, *Helicobacter pylori*, and *Leishmania infantum.*^14^ MEVs represent an innovative platform that integrates multiple immunogenic epitopes to elicit broader and more durable immune protection.^15^ Parallel to advances in vaccinology, computational biology has expanded the opportunities for antimicrobial drug discovery. *In silico* strategies enable the rapid identification of pathogen-specific proteins as potential therapeutic targets, substantially reducing the cost and time compared with traditional approaches.^16^, ^17^ This study aimed to identify novel Omp-derived immunogenic candidates in *S. maltophilia* and design a multi-epitope vaccine using a reverse vaccinology approach. In addition, this study sought to identify potential drug targets, providing insights that may guide the rational development of effective vaccines and new therapeutic strategies against this multidrug-resistant pathogen.

## 2 Materials and methods

### 2.1 Design multi-epitope subunit

#### 2.1.1 Genomic sequence retrieval

**Fig. 1:**
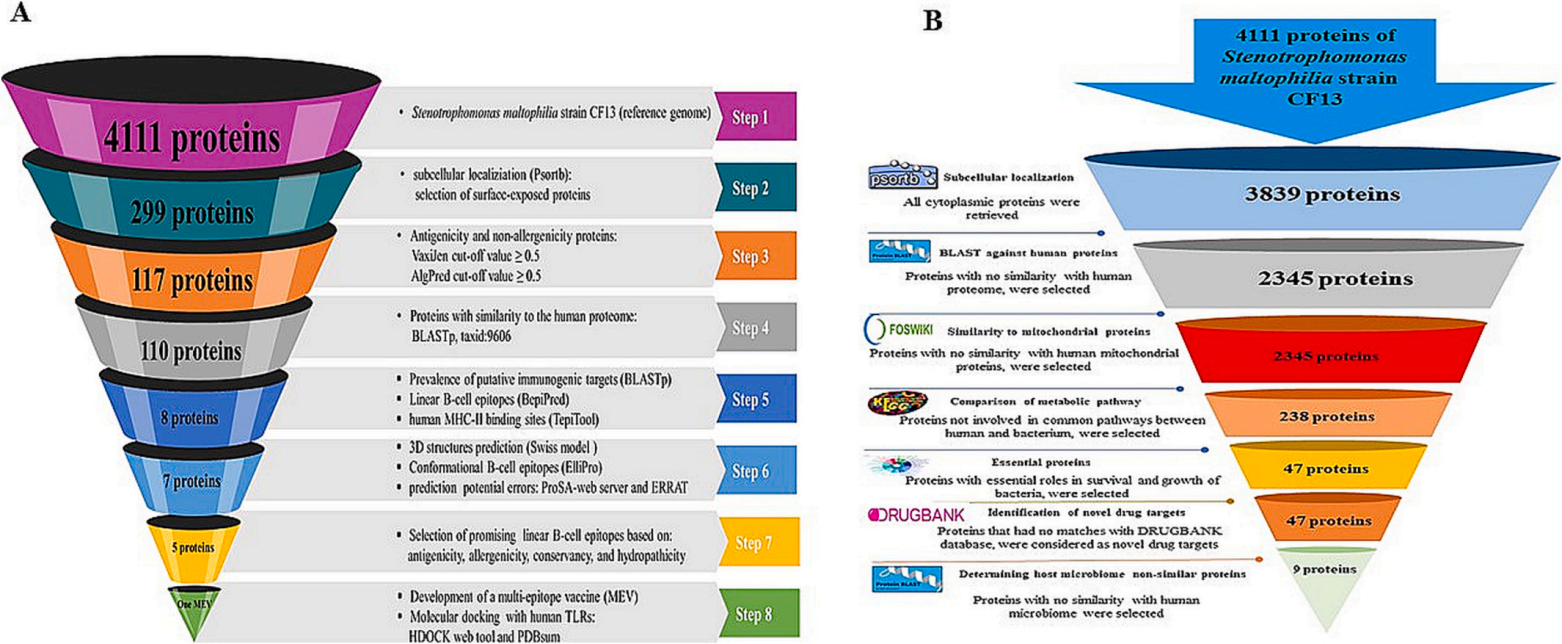
A. The workflow of identification of novel putative immunogenic targets against *S. maltophilia* using a reverse vaccinology approach. All software, databases, web tools, and cut-offs are presented within the workflow. B. The workflow of identification of novel drug targets against *S. maltophilia*. All software, databases, and web tools are presented within the workflow.

A total of 77 *S. maltophilia* strains with complete genome sequences were obtained from the GenBank database (https://www.ncbi.nlm.nih.gov/genbank/) in October 2023 and studied using Bacterial Pan Genomic Analysis (BPGA) software version 1.3 with an identity ≥0.5. The b-parameter of these strains was 0.313. The b-parameter <0.5 indicates that the pan-genome of the studied strains is open. Thus, the ratio of core proteins to pan proteins is low. Given these results, the *S. maltophilia* strain CF13 was selected as the reference strain, and its genomic content was analyzed. This multidrug-resistant isolate was obtained from a sputum sample of a patient with cystic fibrosis in Sydney, Australia 2011.^18^ Thus, the protein-coding sequences were annotated and considered for further analysis in the following steps. Fig. 1 provides an illustration of the workflow used in this study.

#### 2.1.2 Subcellular localization prediction

To identify the proteins' subcellular localization, the PSORTb version 3.0.3 (www.psort.org/psortb/) and TMHMM Server version 2.0 (https://services.healthtech.dtu.dk/service.php?TMHMM-2.0) databases were used. This step retained only extracellular and OMPs of *S. maltophilia* CF13.

#### 2.1.3 Determination of the antigenicity and allergenicity

The antigenicity of the putative immunogenic targets was predicted using the VaxiJen web server v2.0 (http://www.ddg-pharmfac.net/vaxijen/VaxiJen/VaxiJen.html) with a cut-off value of ≥0.5.^19^ Then, the allergenicity of proteins was predicted with the AlgPred 2.0 web tool (https://webs.iiitd.edu.in/raghava/algpred2/batch.html) with a cut-off value ≥0.5.^20^

#### 2.1.4 Determination of the similarity of immunogenic proteins to the proteome of human

The BLASTp tool in the NCBI database (https://blast.ncbi.nlm.nih.gov/) was used to evaluate the homology between the selected immunogenic proteins and human proteome (*Homo sapiens*, taxid:9606). Proteins similar to the human proteome were excluded from the study. PSI-BLAST is more sensitive than standard BLAST in detecting sequence similarities, particularly when sequences are distantly related to the query. Proteins exhibiting ≥30% coverage and ≥ 25% sequence identity were excluded from further analysis.

#### 2.1.5 Prevalence and sequence conservation of immunogenic protein targets among *S. maltophilia* strains

To induce a robust immune response against *S. maltophilia*, the prevalence and conservation of proteins among these strains were assessed. To achieve this, homologs of each immunogenic target in 77 *S. maltophilia* strains were retrieved using BLASTp. Homologous sequences of each protein were aligned using MegaX software.^21^ Proteins with a prevalence of >90% were selected. Sequence conservation of the selected proteins was assessed using the ConSurf web server (https://consurf.tau.ac.il/).^22^

#### 2.1.6 Determining the linear B-cell epitopes and human MHC-II binding sites

Linear B-cell epitopes of all selected immunogenic proteins were identified using the BepiPred v3.0 tool (https://services.healthtech.dtu.dk/service.php?BepiPred-3.0) a cut-off value of ≥0.6.^23^ The ratio of B-cell epitopes to all amino acids in each protein was calculated. Additionally, the TepiTool resource from the IEDB (http://tools.iedb.org/tepitool/help/) was employed to predict human MHC-II binding sites,^24^ with a cut-off for the top 5% of the peptides. Twenty-six of the most prevalent MHC-II alleles predicted MHC-II-binding sites. The ratio of MHC-II binding sites to total amino acids in each protein was calculated.^25^

#### 2.1.7 Prediction and characterization of the tertiary structure and conformational B-cell epitopes

The SWISS-MODEL (https://swissmodel.expasy.org/) was employed to predict the three-dimensional structures of selected vaccine candidate proteins.^26^ Moreover, the ProSA-web server (https://prosa.services.came.sbg.ac.at/prosa.php) and ERRAT (https://saves.mbi.ucla.edu/results?job=1243713&p=errat) were used to show any potential errors in the 3D models and verify their quality.^27^, ^28^ Conformational B-cell epitopes were identified using the ElliPro server (http://tools.iedb.org/ellipro/) using a threshold of ≥0.8. Jmol software was utilized to depict the predicted conformational B-cell epitopes on the surface of each protein, distinguished by different colors.^29^

#### 2.1.8 Predicting physicochemical characteristics of immunogenic targets

Functional classification of the proteins was performed using the VICMpred database (https://webs.iiitd.edu.in/raghava/vicmpred/submission.html).^30^ The physicochemical properties of the proteins were assessed using the Expasy ProtParam tool (https://web.expasy.org/protparam/) to determine their molecular weights and theoretical isoelectric points.

#### 2.1.9 Protein domain search

The Conserved Domain Database (CDD) (https://www.ncbi.nlm.nih.gov/Structure/cdd/cdd.shtml) and EggNOG (http://eggnog5.embl.de/#/app/home) were used to determine the functional domains of the proteins. CDD, an NCBI Entrez query database, annotated the position of conserved domains in protein sequences. In contrast, EggNOG is a hierarchical, functional, and phylogenetically annotated orthology database.^31^, ^32^

#### 2.1.10 Protein-protein interaction using the STRING database

To predict the probable functions of hypothetical proteins, we evaluated their interactions with other *S. maltophilia* proteins using the STRING database (https://string-db.org/). Connection scores of ≥0.5 were taken into consideration.

#### 2.1.11 Prediction and selection of optimal epitopes for vaccine design

B lymphocytes are key players in humoral immunity and produce antibodies that recognize and neutralize pathogens. To identify potential vaccine targets, the selected protein sequences were screened for linear B-cell epitopes using the Kolaskar–Tongaonkar method within the Antibody Epitope Prediction tool on the Immune Epitope Database (IEDB) analysis server (http://tools.iedb.org).^33^ The antigenicity of candidate epitopes was evaluated with the VaxiJen web tool (http://www.ddg-pharmfac.net/vaxijen/VaxiJen/VaxiJen.html) using a threshold of ≥0.5, while allergenicity was assessed *via* AlgPred 2.0 (https://webs.iiitd.edu.in/raghava/algpred2/batch.html) with the same cut-off. Epitope conservation was further analyzed using the IEDB conservancy tool (http://tools.iedb.org/conservancy/). Ultimately, epitopes that were antigenic, non-allergenic, and highly conserved (>90%) were selected as optimal candidates for subunit vaccine development.

#### 2.1.12 Developing a multi-epitope vaccine

Linear B-cell epitopes of the seven shortlisted proteins were predicted using BepiPred v3.0, and their antigenicity, allergenicity, and conservancy were assessed. Antigenic, non-allergenic, highly conserved (>90%), and low hydropathicity score epitopes were selected to construct an MEV against *S. maltophilia*. To enhance immunogenicity, the 50S ribosomal protein L7/L12 from *Mycobacterium tuberculosis*, a TLR-4 agonist, was incorporated as an adjuvant.^34^ The inclusion of adjuvants in MEVs strengthens immune responses and promotes long-lasting immunity.^35^ The selected linear B-cell epitopes were connected using flexible GPGPG linkers, and epitope shuffling was performed to identify the arrangement with the highest antigenicity score. The TLR-4 adjuvant was attached to the N-terminus using a rigid EAAAK linker. The secondary structures of the vaccine were predicted using the PSIPRED server v4.0 (http://bioinf.cs.ucl.ac.uk/psipred/), which employs PSI-BLAST output and two feed-forward neural networks for accurate 2D homology modeling.^36^ Finally, the 3D structure of MEV was modeled using the SWISS-MODEL (https://swissmodel.expasy.org/).^26^ T-cell (MHC-II) binding sites were screened solely to support antigen prioritization and were not included in the MEV construct.

For the allergenicity assessment of the MEV, we employed the AlgPred 2.0 server (https://webs.iiitd.edu.in/raghava/algpred2/batch.html) with a cut-off value ≥0.5. We further analyzed allergenicity to ensure a comprehensive evaluation using AllerTOP v. 2.0 (https://www.ddg-pharmfac.net/AllerTOP/). The outcome of this tool corroborated the non-allergenic nature of the MEV. Based on the combined results from both servers, we concluded that the vaccine construct was likely non-allergenic, minimizing the risk of allergic reactions during vaccination protocols.^20^, ^37^ In the following, the VaxiJen server tool (http://www.ddg-pharmfac.net/vaxijen/VaxiJen/VaxiJen.html) was employed to estimate the antigenicity of MEV. The Protein-Sol server (https://protein-sol.manchester.ac.uk/) was utilized to predict protein solubility so that protein scores over 0.45 are considered soluble.^38^ Next, major physicochemical characteristics of the MEV were predicted using the ExPASy ProtParam web tool (https://web.expasy.org/protparam/). The server estimates a range of parameters, such as molecular weight (MW), isoelectric point (pI), amino acid arrangement, protein half-life in both *in vitro* and *in vivo* conditions, aliphatic index, instability index, and the grand average of hydropathicity (GRAVY) score.^39^ Toll-like receptors (TLRs), particularly TLR-2 and TLR-4, are key components of the innate immune system that detect pathogen-associated molecular patterns (PAMPs) and trigger inflammatory responses. TLR-2 primarily recognizes components of Gram-positive bacteria, as well as certain Gram-negative bacterial products such as lipoteichoic acid and peptidoglycan, thereby mediating immune activation. In contrast, TLR-4 is mainly responsible for detecting lipopolysaccharides (LPS) from Gram-negative bacteria and initiating downstream signaling pathways that result in the production of inflammatory mediators. Activation of TLR-4 is crucial for amplifying the inflammatory response initiated by TLR-2, ensuring a robust immune reaction.^40^, ^41^ This synergistic interaction between TLR-2 and TLR-4 is particularly important for mounting an effective immune defense against *S. maltophilia*. To investigate the ability of MEV to interact with human TLRs, protein-protein docking analysis was performed between MEV and human TLR-2 and TLR-4 using the HDOCK web tool (http://hdock.phys.hust.edu.cn/).^42^ The interactions of the docked complexes were visualized and validated using the PDBsum server (https://www.ebi.ac.uk/thornton-srv/databases/pdbsum/).^43^

#### 2.1.13 Immune response simulations of the vaccine construct

The C-ImmSim tool (https://kraken.iac.rm.cnr.it/C-IMMSIM/) utilizes position-specific scoring matrices to model and predict the strength of immune responses elicited by vaccines over time.^44^ This approach allows researchers to assess how different vaccination schedules influence immune dynamics, providing valuable insights into optimizing immunization strategies and evaluating the potential efficacy of vaccine candidates.

#### 2.1.14 Reverse translation and *in silico* cloning

The Java Codon Adaptation Tool (https://www.jcat.de/) was employed to optimize codon usage and ensure efficient expression of the vaccine construct.^45^ This tool adapts the codon sequence for optimal expression in *Escherichia coli* K12 following established guidelines. Effective expression requires maintaining a GC content between 30% and 70% and achieving a Codon Adaptation Index (CAI) above 0.8.^45^ After codon optimization, the final MEV sequence was subjected to *in silico* cloning using the vector VNTi software to simulate its insertion into an appropriate expression vector.

#### 2.1.15 Molecular dynamics simulation

Molecular dynamics (MD) simulations were performed to assess the stability of the vaccine–TLR-2 and vaccine–TLR4 complexes obtained *via* molecular docking. Simulations were conducted using GROMACS 2018 with Chemistry at Harvard Macromolecular Mechanics 36 (CHARMM36) force field. Each complex was solvated in a dodecahedron box with Transferable Intermolecular Potential 3-Point (TIP3P) water molecules, and 0.15 M sodium chloride (NaCl) was added to neutralize the system and mimic the physiological conditions. Energy minimization was performed using the steepest descent algorithm, followed by 100 ps constant Number, Volume, and Temperature (NVT) equilibration at 298 K (V-rescale thermostat) and 100 ps constant Number, Pressure, and Temperature (NPT) equilibration at 1 bar (Parrinello–Rahman barostat).^46^ Electrostatic interactions were computed using Particle Mesh Ewald (PME) with a 1.0 nm cutoff, and bond constraints were applied using Linear Constraint Solver (LINCS) algorithm. The final production run was conducted for 100 ns, without restraints. Binding free energies were calculated using the Molecular Mechanics/Poisson–Boltzmann Surface Area (MM/PBSA) method with the gmx_MMPBSA tool.^47^

### 2.2 Identification of novel drug targets

#### 2.2.1 Detection of cytoplasmic protein sequences

Using the PSORTb version 3.0 (www.psort.org/psortb/) used above, the cytoplasmic protein sequences of *S. maltophilia* strain CF13 were identified. The workflow of this study is shown in Fig. 1.

#### 2.2.2 Selecting host non-similar protein sequences

The BLASTp tool in the NCBI database (https://blast.ncbi.nlm.nih.gov/) was used to evaluate the homology of the selected proteins with the human proteome (*Homo sapiens*, taxid:9606) using PSI-BLAST. Proteins that were similar to the human proteome were excluded from the study, and the MITOMASTER database (http://mammag.web.uci.edu/twiki/bin/view/Mitomaster) was used to recognize the similarity of the proteins to human mitochondrial proteins.^48^

#### 2.2.3 Detection of essential proteins

The Database of Essential Genes (DEG) was used to analyze bacterial proteins that are not homologous to humans.^49^ The complete set of organism strains from the DEG 15.2 server was selected for analysis. BLASTp was performed with a threshold e-value of 10 and a minimum bit score of 100. Proteins with an identity of 25% or higher and an expectation value of 0–100 were retained because they are considered crucial for the pathogen.^50^

#### 2.2.4 Identification of unique metabolic pathway proteins

The KAAS server (https://www.genome.jp/kaas-bin/kaas_main) of the KEGG database was used to identify the number of vital host non-homologous proteins.^51^ Protein sequences with similar pathways were retrieved for further analysis.

#### 2.2.5 Detecting novel therapeutic targets

To underscore the uniqueness of the selected proteins as drug targets, sequences from earlier stages were scrutinized using the Drugbank server database (https://www.drugbank.ca/structures/search/bonds/sequence).^52^ The presence of targets indicates their druggable potential, whereas the absence of targets highlights the uniqueness of proteins identified as “new targets.” During this process, the predefined settings for each variable remained unchanged.

#### 2.2.6 Determining host microbiome non-similar protein

Many helpful microorganisms within the host organism protect the body from unknown objects. This study aimed to determine whether the distinct metabolic pathway sequences of *S. maltophilia* matched the proteins of these beneficial microorganisms within the host organism. Therefore, the NCBI BLAST server was used for BLAST (see Supplementary File for the gut microbiota).^53^ The proteins with similarities were removed.

#### 2.2.7 Analysis of protein-protein interactions

The STRING 11.5 software server (https://string-db.org) was used to investigate the interaction network of proteins from different drug targets.^54^ high-confidence interactors (score ≥ 0.700) were considered in the protein network. Removal of false-positive and false-negative results. When a target protein is deleted, changes in the number of edges and nodes, which represent connections between proteins and their interactions, illustrate its impact on the biological processes of an organism.^55^

#### 2.2.8 Identify the function of hypothetical proteins based on structural similarity

The BLASTp tool in the NCBI database (https://blast.ncbi.nlm.nih.gov/) was used to investigate the nine hypothetical proteins against the Protein Data Bank (PDB) database to predict their functions.

## 3 Results

### 3.1 Multi-epitope subunit

#### 3.1.1 Identification of surface-exposed, antigenic, and non-allergen proteins

**Fig. 2:**
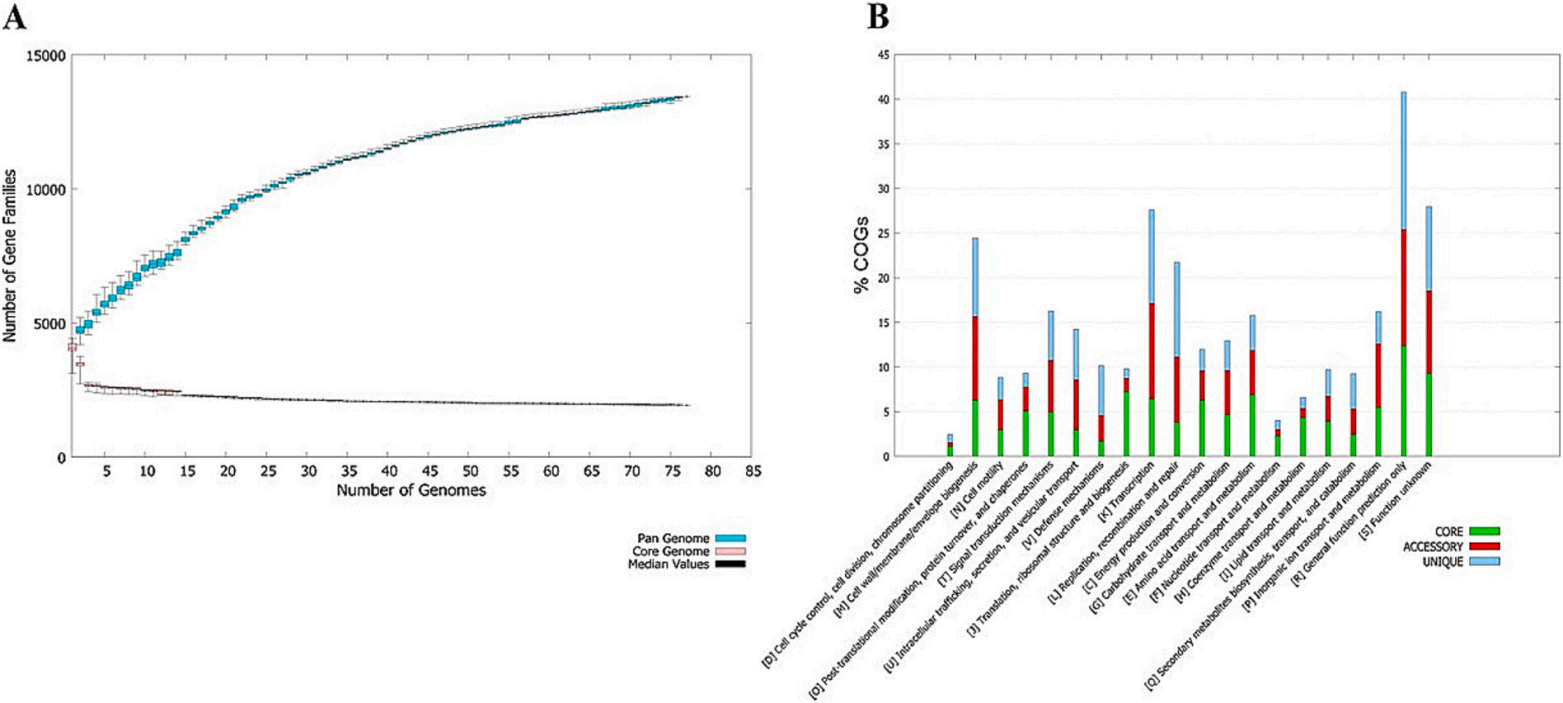
A. The core-pan plot of 77 *S. maltophilia* strains shows 1931 core proteins. B. The analysis of Clusters of Orthologous Groups (COGs) revealed that the majority of core proteins were associated with secondary metabolites biosynthesis, transport and catabolism, translation, ribosomal structure and biogenesis, and cell wall biogenesis.

Among the 77 studied strains, 1931 core proteins were identified with an identity>0.8. The core-pan plot of this dataset is shown in Fig. 2A. Analysis of Clusters of Orthologous Groups (COGs) revealed that the majority of core proteins were associated with secondary metabolite biosynthesis, transport and catabolism, translation, ribosomal structure and biogenesis, and cell wall biogenesis (Fig. 2B). The proteome of the *S. maltophilia* strain CF13 consists of 4111 proteins. Subcellular localization analysis identified 202 surface-exposed proteins. Among them, 200 proteins were antigenic, with a score of ≥0.5. AlgPred analysis identified 117 non-allergenic proteins.

#### 3.1.2 Sequence similarity of proteins with the human proteome

All proteins were analyzed to determine their similarity to *Homo sapiens* (taxid:9606). Finally, seven homologous proteins were excluded, leaving 110 non-homologous proteins.

#### 3.1.3 High prevalence of targets among widespread sequence types (STs)

Seventy-seven *S. maltophilia* isolates with common STs were selected to determine the frequencies of immunogenic candidates. Of the 110 candidate proteins, 102 had a prevalence ≥90%. These highly prevalent proteins were selected for further analysis. Additional information can be found in Supplementary Data 1.

#### 3.1.4 B-cell epitopes and human MHC II binding sites

**Fig. 3:**
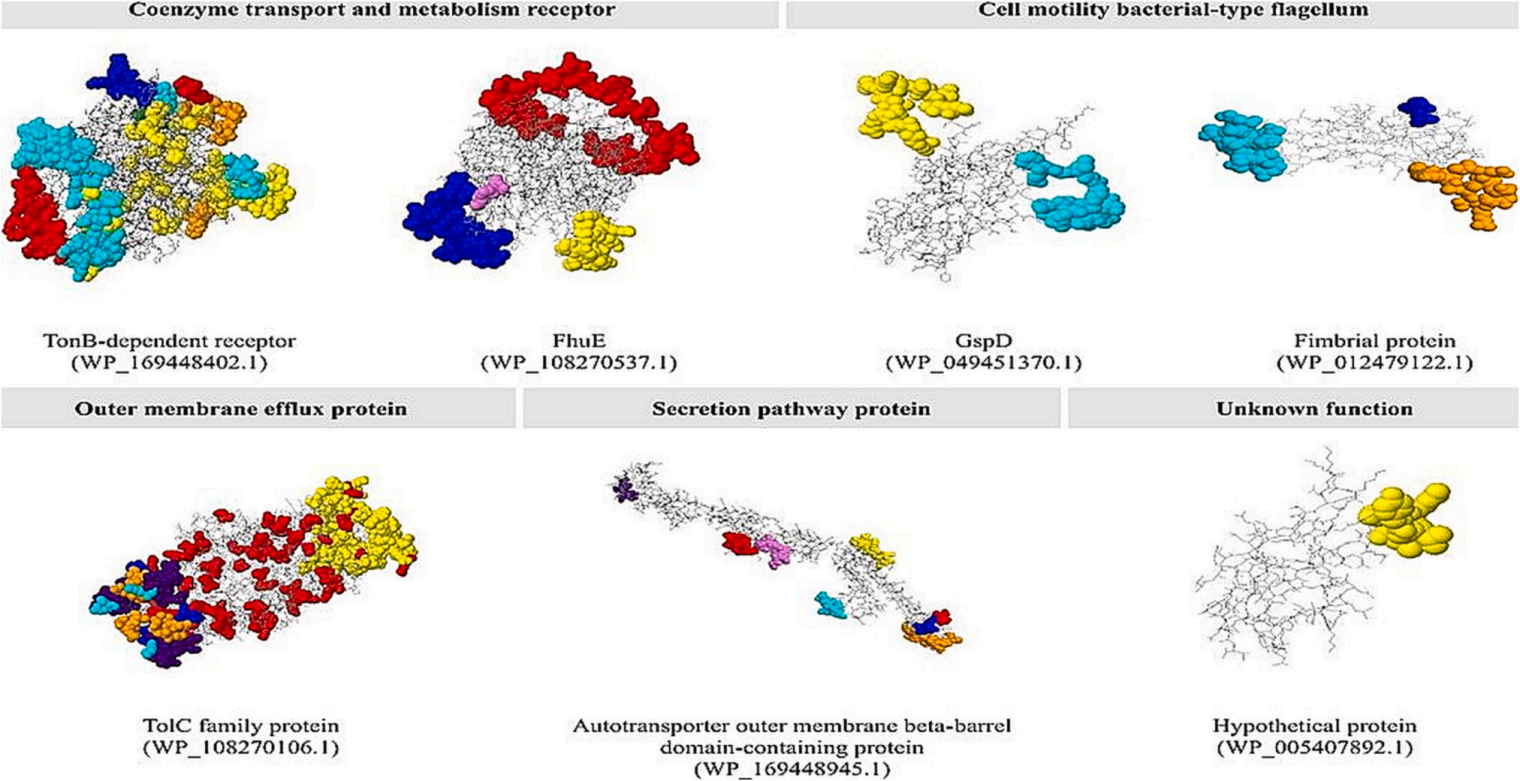
The conformational B-cell epitopes were characterized on the tertiary structure of the proteins using ElliPro and Jmol software. Each colour shows a particular conformational epitope.

**Fig. 4:**
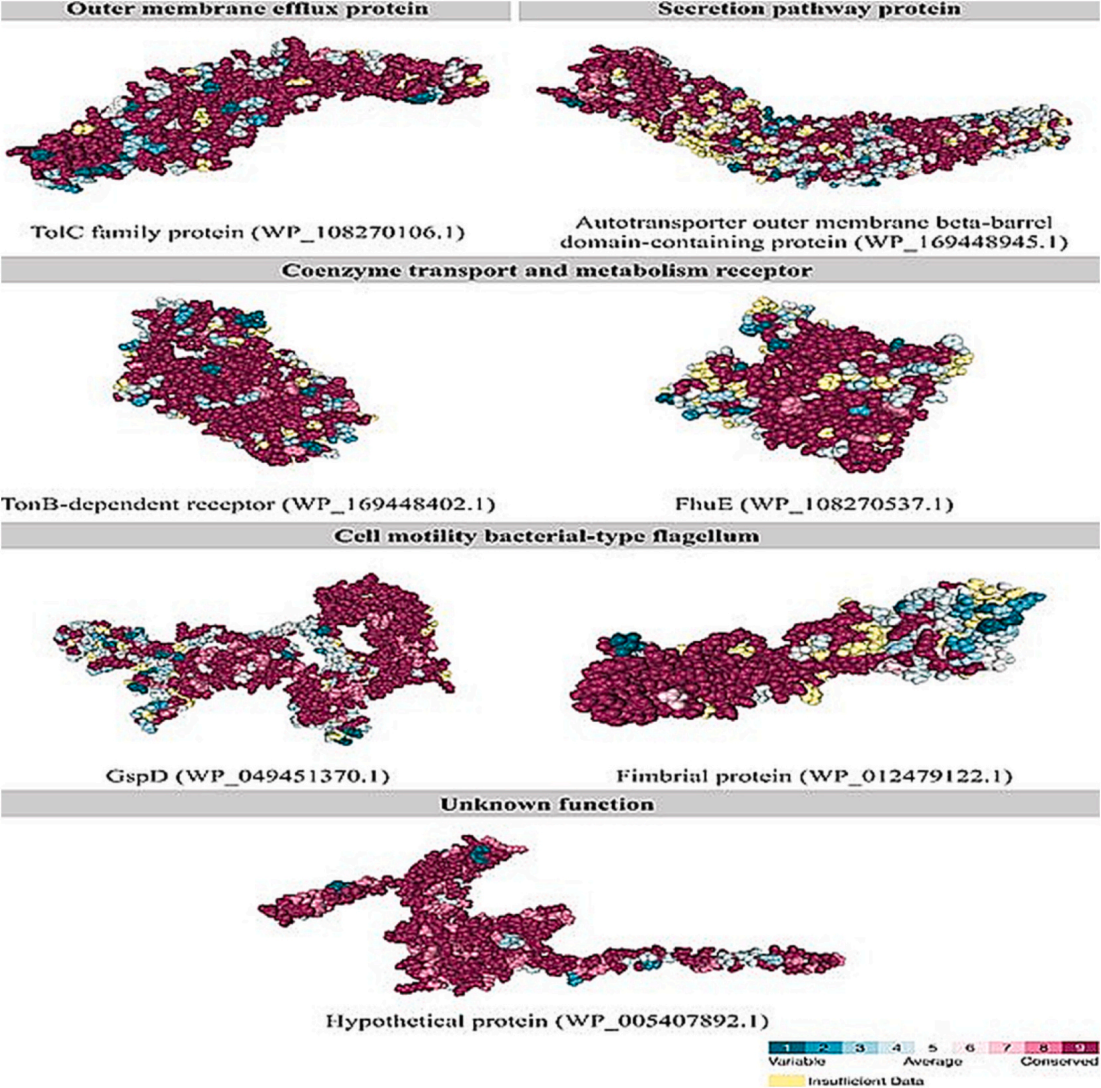
The conservancy of selected proteins among 77 *S. maltophilia* strains using the ConSurf Database. All seven vaccine candidates were almost conserved.

The average ratio of human MHC-II-binding sites to linear B-cell epitopes was 34.61 and 21.22, respectively (Supplementary Data 1). Our analyses revealed that out of 101 proteins, only seven proteins with MHC-II binding sites and linear B-cell epitope ratios above the average were selected. These proteins' tertiary structures were predicted, and their conformational epitopes were determined based on their tertiary structures. The number of linear B-cell epitopes identified for each protein was as follows: TonB-dependent receptor (WP_169448402.1), ten epitopes; Type II secretion system secretin GspD (WP_049451370.1), eight epitopes; TolC family protein (WP_108270106.1), eight epitopes; Ferric-rhodotorulic acid/ferric-coprogen receptor FhuE (WP_108270537), four epitopes; Fimbrial protein (WP_012479122.1), three epitopes; autotransporter outer membrane beta-barrel domain-containing protein (WP_169448945.1), two epitopes; hypothetical protein (WP_005407892.1), one epitope (Fig. 3.). Using the ConSurf Web tool, protein conservation was assessed among 77 strains, revealing that seven proteins were conserved. A schematic representation of the preservation of 3D structures of these proteins is shown in Fig. 4.

#### 3.1.5 Physicochemical properties of potential immunogenic proteins

Their physicochemical characteristics were assessed to evaluate putative immunogenic proteins, and three proteins with molecular weights 110 ≥ kDa were subsequently excluded from further analysis. The functional classes of the remaining proteins were determined using VICMpred. Notably, several proteins were found to be involved in cellular processes, including WP_005407892.1 (hypothetical protein), WP_108270106.1 (TolC family protein), WP_049451370.1 (GspD), WP_108270537.1 (FhuE), and WP_169448402.1 (TonB-dependent receptor). In contrast, WP_012479122.1 (a fimbrial protein) is categorized as a metabolic molecule. WP_169448945.1 (autotransporter beta-barrel domain-containing OMP) was also identified as a virulence factor. Detailed information is shown in Table 1.

**Table 1:** Physicochemical properties of seven putative immunogenic targets against *S. maltophilia*.

Protein (Accession number)	Functional class (score)	Subcellular localization	No. of amino acids	Molecular weight (kDa)	Theoretical pI	EggNOG	CD domain
Hypothetical protein (WP_005407892.1)	Cellular process (0.74)	OMP⁎	344	38.88	9.19	Function unknown	No conserved domain
TolC family protein (WP_108270106.1)	Cellular process (8.74)	OMP	387	43.77	8.94	Cell wall, membrane, envelope biogenesis, Intracellular trafficking, secretion, vesicular transport, Outer membrane efflux protein	Outer membrane protein TolC [Cell wall/membrane/envelope biogenesis]
autotransporter outer membrane beta-barrel domain-containing protein (WP_169448945.1)	Virulence factors (4.3)	OMP	971	103.55	4.7	Cell wall, membrane, envelope biogenesis, Intracellular trafficking, secretion, vesicular transport, surface antigen, secretion pathway protein	outer membrane autotransporter, autotransporter adhesin AIDA-I
TonB-dependent receptor (WP_169448402.1)	Cellular process (−6.28)	OMP	879	99.77	6.37	Coenzyme transport and metabolism receptor, Inorganic ion transport and metabolism Receptor	Porin superfamily. Outer membrane channels
Fimbrial protein (WP_012479122.1)	Metabolism Molecule (0.22)	Fimbrial/ Extracellular	315	36.2	9.21	Coenzyme transport and metabolism receptor	Porin superfamily. Outer membrane channels
GspD (WP_049451370.1)	Cellular process (4.65)	OMP	710	77.3	5.96	Intracellular trafficking, secretion, vesicular transport, Cell motility bacterial-type flagellum	Pilin (type 1 fimbria component protein) [Cell motility]
FhuE (WP_108270537.1)	Cellular process (2.04)	OMP	688	79.14	5.24	Intracellular trafficking, secretion, vesicular transport, Cell motility bacterial-type flagellum	Porin superfamily. Outer membrane channels

⁎ OMP: Outer Membrane Protein.

#### 3.1.6 Conserved domains of immunogenic targets

Conserved domain analysis revealed that TonB-dependent receptors, GspD and FhuE, belong to the porin superfamily, which consists of outer membrane channels. Additionally, the analysis identified the involvement of TolC family proteins and the autotransporter beta-barrel domain-containing OMP in various cellular processes, such as cell wall, membrane, and envelope biogenesis, as well as intracellular trafficking, secretion, and vesicular transport. Fimbrial protein (WP_012479122.1) is associated with coenzyme transport and metabolic receptors. However, no conserved domains were found in this hypothetical protein (WP_005407892.1). Detailed results from the Conserved Domain Database (CDD) and EggNOG analysis for immunogenic proteins are shown in Table 1.

#### 3.1.7 Protein-protein interaction networks

**Fig. 5:**
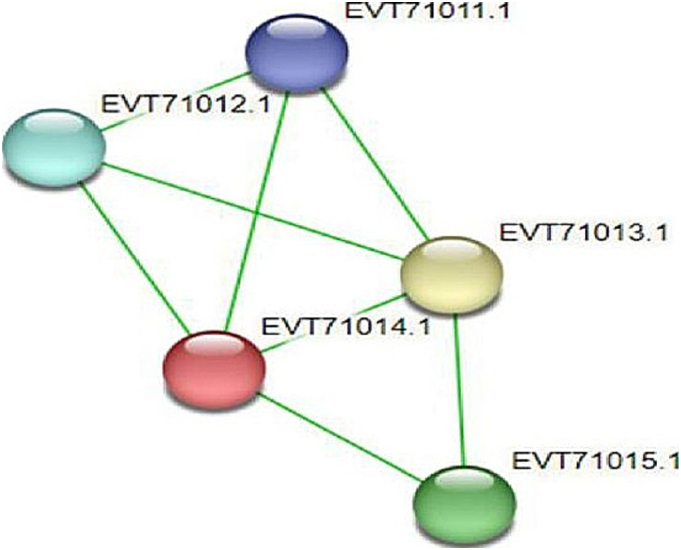
Protein-protein interaction of hypothetical protein (WP_005407892.1) with other proteins of *S. maltophilia* using STRING database. This protein (red ball) has neighborhood relationship with two other hypothetical proteins (EVT71015.1 and EVT71013.1), an RNA polymerase subunit sigma-24 (EVT71012.1), and an RNA signal recognition particle (EVT71011.1). Predicted Interactions: (Green: gene neighborhood). (For interpretation of the references to colour in this figure legend, the reader is referred to the web version of this article.)

STRING analysis of the hypothetical protein (WP_005407892.1) revealed several protein interactions with the sigma-24 RNA polymerase subunit. These subunits are associated with all features of transcription initiation, including promoter location, promoter melting, initiation of RNA synthesis, abortive initiation, and promoter escape.^56^ Additionally, RNA signal recognition performs specific roles in co-translational translocation (primarily by incorporating proteins into the inner membrane) (Fig. 5).

#### 3.1.8 Multi-epitope vaccine (MEV) against *S. maltophilia*

**Fig. 6:**
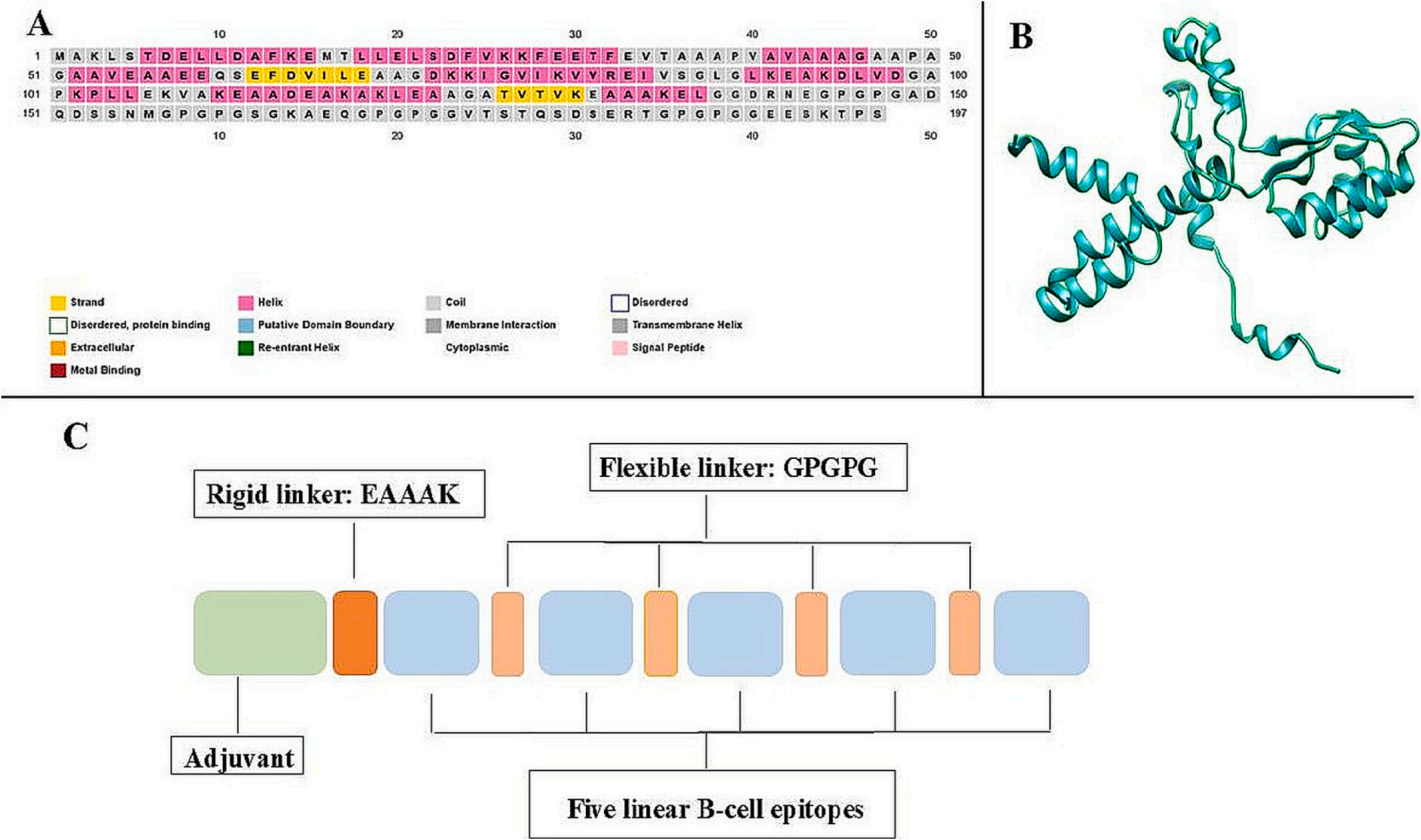
A. The secondary structure of MEV was predicted using the PSIPRED server B. The tertiary structure of MEV was predicted using SWISS-MODEL. C. The schematic representation of MEV components' arrangement. Linear B-cell epitopes were joined together using GPGPG flexible linkers. 50S ribosomal protein L7/L12 of *M. tuberculosis* was attached to the N-terminal of the construct as an adjuvant.

The secondary structure of the vaccine was predicted using the PSIPRED 4.0 server, which indicated that coil regions were the most abundant (51.26%), followed by α-helices (42.63%) and β-strands (6.09%) (Fig. 6A). The MEV was generated using the following five immunogenic epitopes: ADQDSSNM (epitope No. 1), SGKAEQ (Epitope No. 2), GEESKTPS (Epitope No. 3), GVTSTQSDSERT (epitope No. 4), and ELGGDRNE (epitope No. 5). Epitope shuffling was performed, and 120 unique arrangements were created (Fig. 6B). The arrangement with the highest antigenicity (score = 1.8811) was selected to design the MEV. Linear B-cell epitopes were joined together using GPGPG flexible linkers. 50S ribosomal protein L7/L12 of *M. tuberculosis* was attached to the N-terminus of the construct as an adjuvant and TLR-4 agonist. Detailed information on epitope evaluation and MEV design is presented in Supplementary Data 2.

**Fig. 7:**
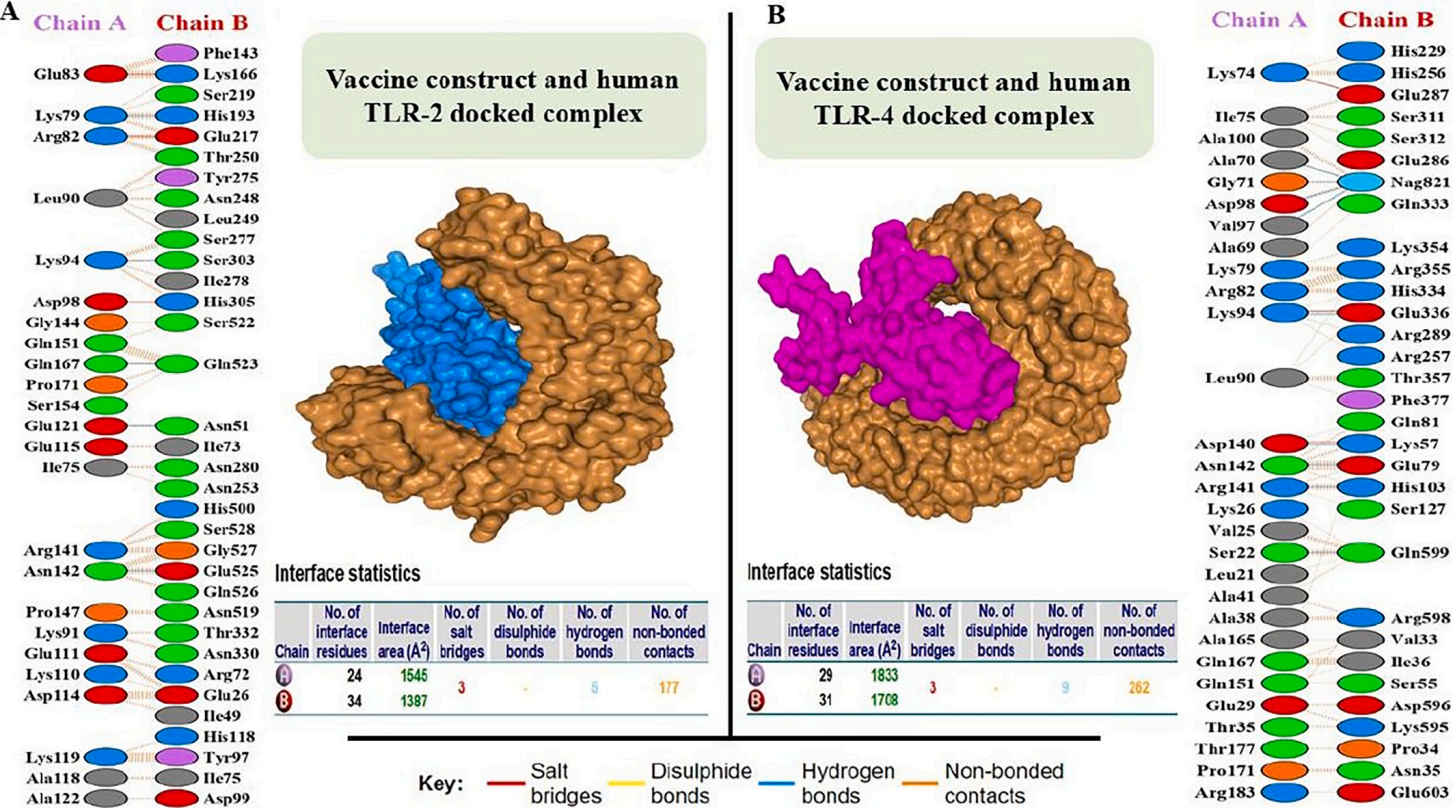
A. The docked complex of human TLR-2 (brown) and MEV (blue). A total of 24 and 34 residues of MEV and TLR-2 were interacted. The number of salt bridges, disulfide, and hydrogen bonds was three, zero, and five, respectively. B. The docked complex of human TLR-4 (brown) and MEV (pink). A total of 29 and 31 residues of MEV and TLR-4 were interacted. The number of salt bridges, disulfide, and hydrogen bonds was three, zero, and nine, respectively. (For interpretation of the references to colour in this figure legend, the reader is referred to the web version of this article.)

A schematic representation of this construct is shown in the Fig. 6C. Finally, the construct was docked with human TLRs. PDBsum analysis revealed that 24 residues of the MEV and 34 residues of TLR-2 participated in the interaction. The complex formed three salt bridges, five hydrogen bonds, and 177 non-bonded contacts, with a docking score of −200.77, a confidence score of 0.7341, and a ligand RMSD of 65.15 Å (Fig. 7A). Furthermore, 29 and 31 MEV and human TLR-4 residues are involved in molecular interactions. Moreover, three salt bridges and nine hydrogen bonds, and 262 non-bonded contacts are formed (docking score: −215.42, Confidence Score: 0.7782, and Ligand RMSD (Å): 80.83) (Fig. 7B). The antigenicity score of MEV predicted by the VaxiJen server was 0.8529. All were approved as non-allergenic, as substantiated by AllerTOP v2.0. The vaccine had the highest solubility score (0.994). MEV had a low MW of 19.758 kDa and extreme thermotolerance, as demonstrated by its high aliphatic index (74.47). The theoretical pI of the MEV was 4.48. The MEV was recognized as hydrophilic because it had a negative GRAVY score (−0.329). As the instability index of the vaccine candidate in the present study was 27.45, which is below 40, they were assigned as “stable” polypeptides. MEV half-life was estimated at 30 h for mammalian reticulocytes *in vitro* and > 10 h for *E. coli in vivo* (Table 2).

**Table 2:** Docking interactions of MEV with TLR-2 and TLR-4, and predicted physicochemical and immunological properties of the vaccine candidate.

Parameter	MEV–TLR-2 Docking	MEV–TLR-4 Docking	Vaccine Candidate Properties
Residues Involved (MEV)	24	29	–
Residues Involved (TLR)	34	31	–
Salt Bridges	3	3	–
Hydrogen Bonds	5	9	–
Antigenicity (VaxiJen)	–	–	0.8529
Allergenicity (AllerTOP v2.0)	–	–	Non-allergenic
Solubility Score	–	–	0.994
Molecular Weight (MW)	–	–	19.758 kDa
Aliphatic Index	–	–	74.47 (extreme thermotolerance)
Theoretical pI	–	–	4.48
GRAVY Score	–	–	−0.329 (hydrophilic)
Instability Index	–	–	27.45 (stable, <40)
Estimated Half-life	–	–	30 h (mammalian reticulocytes, *in vitro*); >10 h (*E. coli*, *in vivo*)

#### 3.1.9 *In silico* cloning of MEV

**Fig. 8:**
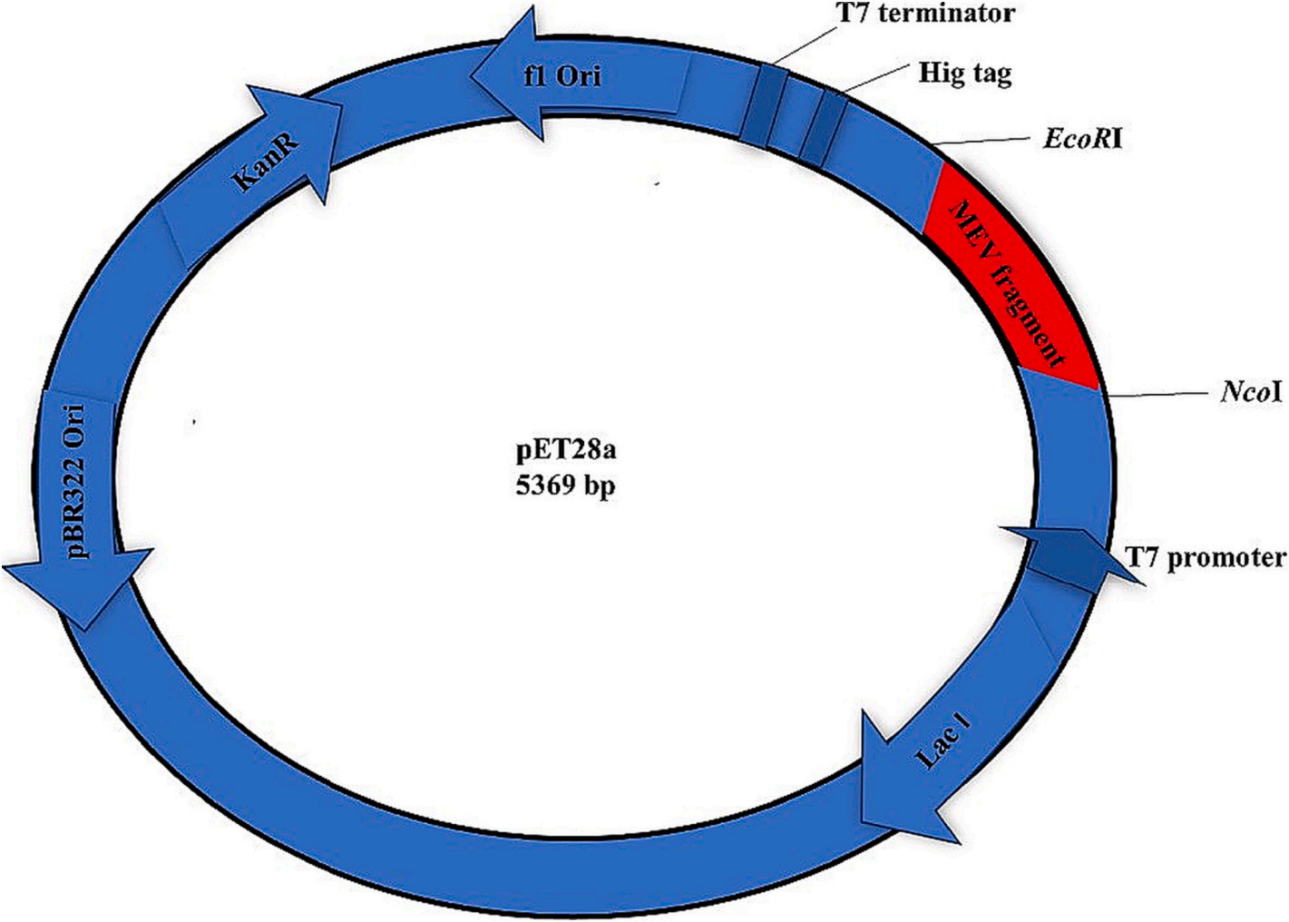
*In silico* restriction cloning of the reverse-translated MEV candidate fragment into the pET28a expression plasmid vector. The red segment between *EcoRI* and *NcoI* represents the insertion site of the vaccine candidate fragment. (For interpretation of the references to colour in this figure legend, the reader is referred to the web version of this article.)

To maximize expression and processivity in *E. coli*, the MEV sequence was reverse-translated according to the *E. coli* K12 codon usage. The Codon Adaptation Index (CAI) was calculated as 1.0, indicating optimal codon compatibility with the host cells. The sequence also displayed a GC content of 51.77%, which is within the favorable 30–70% range for efficient expression in *E. coli*. The optimized vaccine sequence was then cloned into a high-expression pET28a plasmid vector using SnapGene software (Fig. 8).

#### 3.1.10 Immune simulation of MEV

**Fig. 9:**
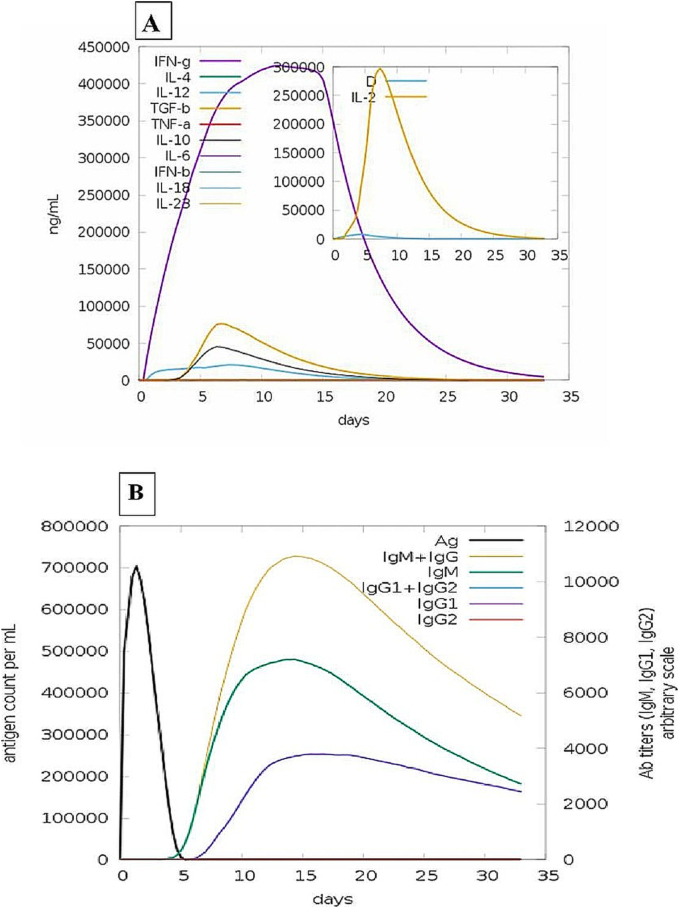
Predicted Immune Response to MEV Vaccine Construct Using the C-ImmSim Tool. A. Cytokine profile showing rapid induction of pro-inflammatory and regulatory cytokines, with IFN-γ displaying the strongest response, peaking at days 10–15, while IL-2 exhibited a sharp transient peak consistent with early T cell activation. B. Antigen clearance and antibody dynamics, with antigen levels peaking at days 3–4 followed by clearance. IgM appeared first, peaking around day 10, then shifted to IgG subclasses (IgG1, IgG2), indicating class switching and a durable adaptive immune response.

Immune simulations revealed the rapid induction of both pro-inflammatory and regulatory cytokines. IFN-γ exhibited the most pronounced increase, peaking between days 10 and 15 before gradually declining. Other cytokines, including IL-2, IL-4, IL-6, IL-10, IL-12, TNF-α, TGF-β, IL-18, IFN-β, and IL-28, were also elevated during the early phase, each displaying distinct magnitudes and kinetics of change. Notably, IL-2 showed a sharp, transient peak, consistent with its role in early T-cell activation. Overall, the cytokine profile reflected a coordinated innate and adaptive immune response involving both Th1- and Th2-associated mediators (Fig. 9A). Simulations further illustrated the dynamics of antigen clearance and the production of antibodies. The antigen level rose rapidly during the initial days post-exposure, peaking around days 3–4, followed by a sharp decline due to immune-mediated clearance. Correspondingly, antibody responses were initiated, with IgM appearing first and peaking around day 10, followed by class switching to IgG subclasses (IgG1 and IgG2). The combined IgM and IgG response demonstrated strong and sustained elevation, with IgG1 and IgG2 titers progressively increasing and remaining higher than IgM, indicating effective class switching and the development of durable adaptive immunity (Fig. 9B). Collectively, these results suggest that the antigen induces potent cytokine-mediated immune activation, promotes efficient antigen clearance, and elicits robust, long-lasting antibody responses, highlighting its strong immunogenic potential and capacity to confer prolonged protective immunity.

#### 3.1.11 Molecular dynamics simulation

**Fig. 10:**
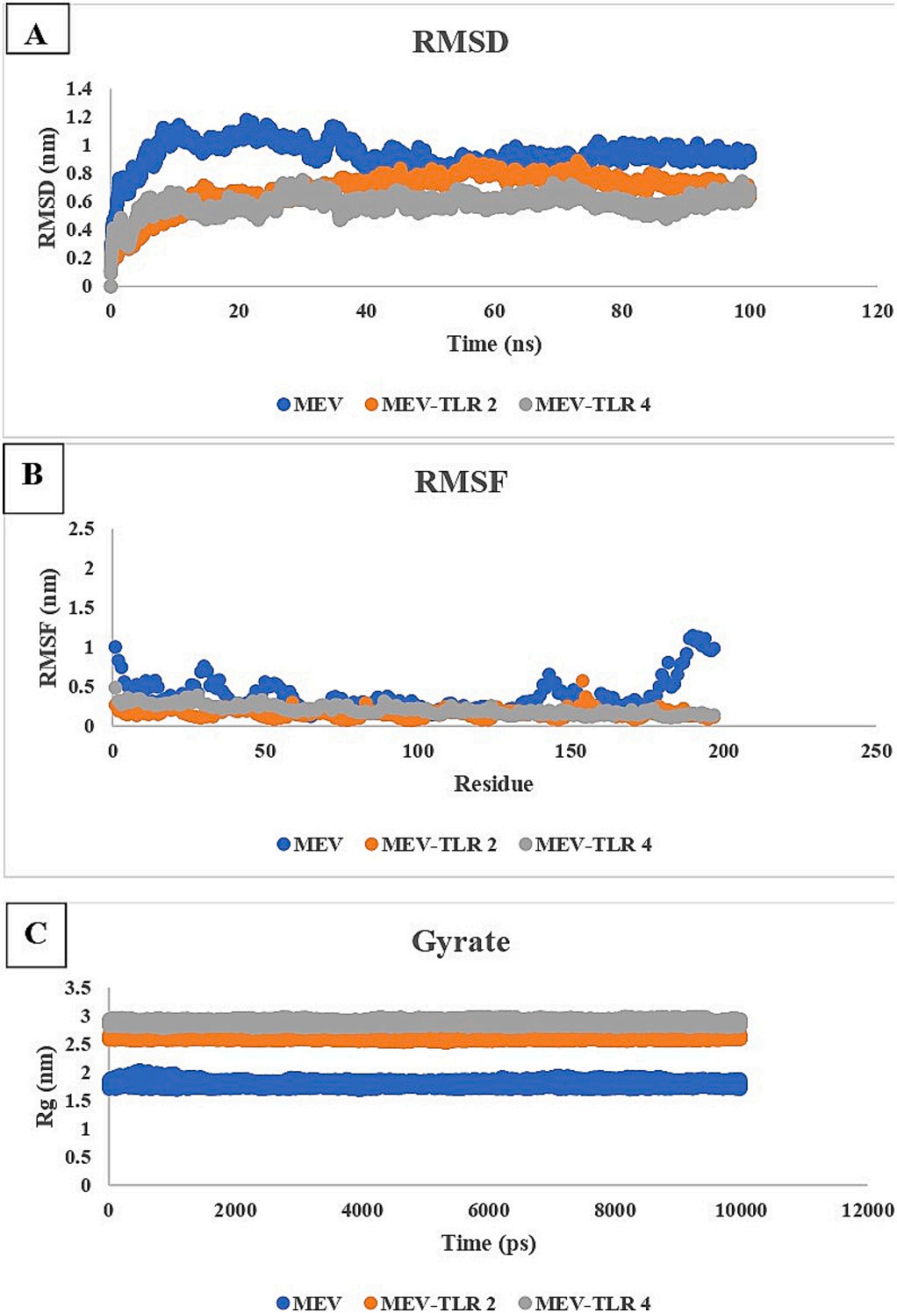
Molecular dynamics simulations of MEV and its Complexes with TLR-2 and TLR-4. (A) RMSD plot showing the stability of the MEV molecule and the MEV_TLR-2 and MEV_TLR-4 complexes throughout the simulation, with equilibrium RMSD values indicating stable interactions. (B) RMSF plot illustrating residue flexibility, where higher RMSF values represent more flexible regions and lower values denote rigidity across the MEV, MEV_TLR-2, and MEV_TLR-4 systems. (C) Radius of gyration (Rg) plot reflecting the shape and compactness of each system over time. Stable Rg values indicate consistent molecular shapes, while fluctuations indicate conformational changes. These simulations offer insights into the stability, flexibility, and structural evolution of MEV molecules and their interactions with TLR receptors under cytosolic conditions.

Molecular dynamics (MD) simulations were conducted to evaluate the stability and flexibility of the MEV construct and its complexes with TLR-2 and TLR-4 receptors. The Root Mean Square Deviation (RMSD) profiles indicated that all systems achieved equilibrium within 20 ns, confirming overall structural stability. The MEV–TLR-4 complex showed the lowest deviation (∼0.6 nm), suggesting the strongest conformational stability upon binding (Fig. 10A).

The Root Mean Square Fluctuation (RMSF) analysis revealed limited residue fluctuations, particularly reduced flexibility in the binding interfaces of the MEV–TLR-2 and MEV–TLR-4 complexes, supporting stable protein–protein interactions (Fig. 10B).

Radius of gyration (Rg) values remained constant throughout the simulation, indicating compact and stable conformations. Collectively, these findings demonstrate that MEV forms highly stable and tightly bound complexes with both TLR-2 and TLR-4, reinforcing its potential as a robust immunogenic vaccine candidate (Fig. 10C).

### 3.2 Identification of novel drug targets

#### 3.2.1 Detection of cytoplasmic protein sequences proteins with no similarities to the host

All proteins in the reference strain were subjected to PSORTb and CELLO. Finally, 3839 proteins were considered to be cytoplasmic. Using PSI-BLAST, 2345 proteins were found to be similar to human proteins. Thus, 1494 proteins identical to the human proteome were excluded. None of the proteins were similar to human mitochondrial proteins.

#### 3.2.2 Identification of unique metabolic pathway proteins

As a result of a BLAST search using the KAAS database, 1375 of the 2345 non-homologous proteins were found to be involved in metabolic pathways and were excluded. Two hundred thirty-eight non-homologous proteins were retrieved for subsequent analysis.

#### 3.2.3 Selecting essential protein sequences

Antibacterial substances are often designed to interact with vital genes. Therefore, essential proteins are considered the most effective therapeutic targets.^57^ Using a dataset of crucial bacterial antigens maintained at the DEG 15.2 site, 47 proteins demonstrated significant hits. Assessment of bacterial antigens is essential for *S. maltophilia* survival. Important genetic components unique to microbes can assist as therapeutic agents for this species.^58^

#### 3.2.4 Identify novel drug targets

All 47 *S. maltophilia* proteins had no similarities to the targets of the FDA-approved or experimental drugs in the DrugBank database and were considered.

#### 3.2.5 Determining host microbiome non-similar proteins

Finally, nine pathogen proteins were identified through the microbiome BLAST as having a resemblance that is not similar to microbial flora in humans. As a result, they were proposed to be the exclusive drug targets of the pathogen (Table 3).

**Table 3:** Identification of the availability of the nine novel drug targets.

No.	Accession number	Protein	Gut microbiota BLAST	PBD target	EggNOG
1	WP_053451664.1	Hypothetical protein	Neg	N	No orthologs found
2	WP_158230916.1	Hypothetical protein	Neg	N	Function unknown
3	WP_005410107.1	Hypothetical protein	Neg	N	Function unknown
4	WP_005413792.1	Hypothetical protein	Neg	N	Function unknown
5	WP_005409784.1	Hypothetical protein	Neg	N	Function unknown
6	WP_224482002.1	Hypothetical protein	Neg	N	No orthologs found
7	WP_169448580.1	Hypothetical protein	Neg	N	Function unknown
8	WP_005409247.1	Hypothetical protein	Neg	N	Function unknown
9	WP_019338257.1	Hypothetical protein	Neg	N	No orthologs found

Neg: negative; N: Not detected.

#### 3.2.6 Hypothetical proteins

Based on the CDD and EggNOG results, the function of the nine drugs targets proteins detected WP_053451664.1, WP_158230916.1, WP_005410107.1, WP_005413792.1, WP_005409784.1, WP_224482002.1, WP_169448580.1, WP_005409247.1, and WP_019338257.1 have no conserved domains and were detected as hypothetical proteins. According to BLAST, the PDB database did not detect any function of these nine proteins (Table 3).

#### 3.2.7 Protein-protein interactions

Results from the STRING database showed that the protein with accession number WP_005410107.1 has neighborhood interactions with NAD(*P*)H dehydrogenase (Smal_2429) and GCN5-related *N*-acetyltransferase (Smal_2430) (Fig. 8). No other proteins were identified in this database.

## 4 Discussion

The escalating emergence of MDR *S. maltophilia* poses a significant hazard to healthcare settings, causing severe infections with diminished responsiveness to traditional antibiotics. Vaccination is a promising alternative for disease control, offering potential long-term protection and reducing reliance on antibiotics.^3^, ^59^ Although traditional vaccines have proven effective against many pathogens, developing vaccines against *S. maltophilia* has presented significant challenges. Reverse vaccinology offers a promising avenue for targeted vaccine development with potential advantages regarding safety, stability, and engineering.^60^ In this study, we adopted a reverse vaccinology approach to address the critical unmet need for an effective *S. maltophilia* vaccine by identifying a promising epitope-based vaccine candidate with the potential to overcome the restrictions of traditional vaccine development strategies.

The open pan-core genome of *S. maltophilia* meticulously selected a reference strain for reverse vaccinology. Consequently, considering two critical criteria, we identified the *S. maltophilia* strain CF13 as a pertinent reference. As the first criterion, CF13 was isolated from a patient with cystic fibrosis. Alignment with a targeted patient population for vaccine development strengthens the translational potential of this strain. CF13 exhibited multidrug resistance as the second criterion, including resistance to last-resort antibiotics. This highlights its clinical significance and underscores the crucial need for alternative intervention strategies, such as vaccines, to combat this increasingly problematic pathogen.

This study highlights the TolC protein family (WP_108270106.1) as a promising vaccine candidate for *S. maltophilia*. The involvement of proteins in multidrug efflux, a known contributor to antibiotic resistance, supports this hypothesis.^61^ We are also encouraged by the results of studies on other pathogens. TolC plays a vital role in the TolCsm efflux pump system, which confers aminoglycoside resistance to *S. maltophilia.*^62^ Furthermore, studies on *Shigella sonnei* and *Vibrio spp.* have identified TolC as a probable vaccine target.^63^, ^64^ This aligns with our findings and strengthens the argument that TolC is a valuable target for vaccine development *against S. maltophilia*. Our study complements the findings of Xu et al.,^65^ who demonstrated the potential of TolC and associated proteins, such as Smlt4123, for vaccine development.

Our study identified the autotransporter beta-barrel OMP (WP_169448945.1) as a highly promising vaccine candidate, reinforcing previous findings by Xu et al. that OmpA can elicit protective immunity against *S. maltophilia.*^66^ This convergence underscores the pivotal role of OMPs as primary targets for host immune recognition and suggests that leveraging multiple OMPs, including OmpA and the autotransporter beta-barrel protein, could enhance vaccine efficacy. Future studies should rigorously evaluate the individual and combined immunogenic potentials of these antigens, including TolC, to optimize protective responses. Furthermore, GspD (WP_108270537.1), a Type II secretion system porin, is a compelling target because of its outer membrane localization and accessibility to antibody-mediated neutralization.^67^ Similarly, the fimbrial protein (WP_012479122.1), a type 1 fimbrial adhesin involved in motility and biofilm formation, presents a surface-exposed epitope suitable for humoral immune targeting. Collectively, these findings highlight the strategic value of surface-exposed proteins in eliciting robust protective immunity and provide a rational foundation for designing multi-antigen vaccines against this multidrug-resistant pathogen.

Furthermore, the association of this protein with opportunistic infections, particularly in immunocompromised individuals, underscores its potential significance in preventing severe outcomes in vulnerable populations.^68^

Both FhuE (WP_049451370.1) and the TonB-dependent receptor (TBDR) (WP_169448402.1) have been identified as promising candidates for *S. maltophilia* vaccine development. Their roles in iron acquisition and outer membrane localization make them attractive targets for disrupting bacterial processes and eliciting effective immune responses.^69^, ^70^ By incorporating these targets, our vaccine design aims to maximize efficacy against the target infection.

We identified a hypothetical protein (WP_005407892.1) that interacted intriguingly with RNA polymerase subunit sigma-24. This suggests a vital role within the cellular machinery of *S. maltophilia*, rendering it an attractive candidate for vaccine development.^71^

Our multi-epitope vaccine design incorporated seven meticulously selected proteins (WP_108270537.1, WP_049451370.1, WP_012479122.1, WP_169448402.1, WP_108270106.1, WP_169448945.1, and WP_005407892.1) with a focus on surface-exposed antigenic epitopes. Our vaccine design prioritizes incorporating immunogenic epitopes, aiming to maximize the vaccine's efficacy against the target infection. MEV has several key features that create a strong immune response. We strategically selected 50S ribosomal protein L7/L12 from *M. tuberculosis* as an adjuvant.^72^ This protein serves as a well-known activator of TLR-4, which is critical for triggering innate and adaptive immune responses. This synergistically amplifies the effect of B-cell epitopes within the MEV, leading to a more robust overall immune defense. This approach aligns with established vaccine design strategies, and the specific targeting of TLR-4 can potentially direct the immune response toward cellular pathways.

Furthermore, within the MEV, individual B-cell epitopes are strategically connected by a flexible linker called GPGPG. This linker mimics proteins' natural spacing and conformational flexibility, effectively ensuring the optimal positioning of B-cell receptors to recognize and bind to each epitope without interfering with it.^73^ This maximized interaction significantly contributed to the overall antigenicity and effectiveness of MEV. Finally, docking analysis revealed promising interactions of the MEV with both TLR-2 and TLR-4 receptors. This finding reinforces the potential of our adjuvant to stimulate robust immune responses *via* these key immune pathways.

Ezaj et al. adopted an in-depth characterization approach, focusing on 24 hypothetical proteins (HPs). However, this approach provides detailed insights into the 24 specific proteins, potential virulence factors, and immune targets and lacks a dedicated vaccine design.^74^ In contrast, our study focused on designing and developing an MEV against *S. maltophilia* by incorporating five B-cell epitopes of seven proteins. Furthermore, we evaluated the potential interactions between MEV and human TLRs. Although distinct in methodologies, these studies offer valuable complementary insights.

The designed MEV construct demonstrated stable and reproducible interactions with TLR-2 and TLR-4, as confirmed by molecular docking and dynamics simulations.^75^ These interactions indicate that MEV can effectively activate both innate and adaptive immunity, suggesting its potential to elicit a robust, multifaceted immune response while maintaining safety for human use. Overall, the *in silico* simulations support the potential of this vaccine to induce durable immunity against *S. maltophilia* while maintaining a favorable safety profile for human use. The *in silico* immune simulations of our MEV closely reflected the immune dynamics observed in patients with *CF* and murine models of *S. maltophilia* infection. The vaccine elicited rapid and robust induction of pro-inflammatory and regulatory cytokines, with IFN-γ peaking between days 10 and 15 and an early IL-2 surge, indicative of efficient T-cell priming. Simultaneous elevation of IL-4, IL-6, IL-10, IL-12, TNF-α, and other cytokines demonstrates a well-coordinated Th1/Th2 response, consistent with experimental reports of strong IL-6, IL-12, IFN-γ, and TNF-α responses, alongside early chemokine activation.^76^ Antigen clearance and humoral responses further confirmed effective immunity. Initial IgM production was followed by sustained IgG1 and IgG2 titers, reflecting successful class switching and durable, adaptive protection. These outcomes parallel the clinical findings in patients with *CF*, where chronic *S. maltophilia* colonization drives robust antibody responses and coordinated innate-adaptive interactions involving macrophages, neutrophils, Th1/Th17 cells, and B lymphocytes.^66^, ^76^, ^77^, ^78^, ^79^, ^80^ Collectively, these results underscore the potent immunogenicity of MEV and its promise as a prophylactic strategy against this opportunistic pathogen.

Nine novel proteins that do not resemble any existing drug targets or proteins from the human microbiome have been identified. This makes them suitable candidates for further research. Additionally, the analysis revealed potential interactions between protein WP_005410107.1 and two neighboring proteins involved in cellular metabolism. This suggests that targeting this protein may have a broader impact on the physiology of *S. maltophilia*. Further research is needed to confirm these interactions and understand their functional significance.

A study identified two essential enzymes, Acyl-[acyl-carrier-protein]—UDP-N acetyl glucosamine O-acyltransferase and D-alanine-D-alanine ligase, as potential drug targets. Eight natural product inhibitors with promising binding interactions and safety profiles were identified. However, the limited number of identified targets restricts treatment options and requires further validation through *in vitro* and *in vivo* experiments to confirm efficacy and biocompatibility.^81^ Our study employed a broader bioinformatics approach, uncovering nine novel essential proteins with no similarity to existing drug targets or human microbiome proteins. These unique proteins are desirable candidates for future research.

Additionally, WP_005410107.1 interacted with metabolic neighbors, suggesting its potential to disrupt vital pathways. Although validation and functional studies are needed, these findings highlight opportunities to target critical cellular processes within bacteria. By combining this broader approach with established methods that target essential proteins, future research can lead to more effective therapies against *S. maltophilia*.

## 5 Conclusion

In this study, we explored an MEV against *S. maltophilia* using reverse vaccinology. Seven promising target proteins were identified using this strategy. The designed MEV incorporated five putative immunogenic B-cell epitopes derived from these proteins, fostering broad immune coverage and potentially addressing the challenges of strain variation. Moreover, subtractive genomics led to the identification of nine novel drug targets in *S. maltophilia* as a complementary approach. This approach minimizes potential side effects by selecting proteins with no similarity to human proteins. Our findings from the current study might form the basis for immunization and drug discovery against this multidrug-resistant pathogen. However, further *in vitro* and *in vivo* experiments are required to assess the safety, immunoreactivity, and efficacy of the MEV and drug targets.

## CRediT authorship contribution statement

**Safoura Moradkasani:** Writing – review & editing, Writing – original draft, Visualization. **Narjes Noori Goodarzi:** Writing – review & editing. **Masoumeh Beig:** Writing – review & editing. **Mohammad Sholeh:** Software. **Sepideh Fereshteh:** Writing – review & editing. **Mahshid Khazani Asforooshani:** Writing – review & editing. **Behzad Shahbazi:** Methodology, Software. **Shahla Shahbazi:** Writing – review & editing. **Farzad Badmasti:** Writing – review & editing, Supervision, Project administration, Investigation.

## **Ethical statement**

This article did not include any human or animal studies conducted by the authors.

## Funding

No funding was received for this study.

## Declaration of competing interest

The authors declare that they have no known competing financial interests or personal relationships that could have appeared to influence the work reported in this paper.

## Appendix A Supplementary data

Supplementary material 1 Supplementary Data 1: Contains detailed information on the gut microbiota.

Supplementary material 2 Supplementary Data 2: Provides comprehensive details on epitope evaluation and multi-epitope vaccine (MEV) design.
