# Supplementary material for: Integrated immunoinformatics for the design of novel multi-epitope vaccine and identification of new drug targets against Stenotrophomonas maltophilia, a multidrug-resistant superbug
Source: J Genet Eng Biotechnol. 2026 Jul 28;24(3):100772. doi: 10.1016/j.jgeb.2026.100772 (PMC13449788; doi:10.1016/j.jgeb.2026.100772)
Supplement: Supplementary file 1 [file mmc1.docx]

**Supplementary Data 1.** Gut microbiota strains

1. *Actinomyces odontolyticus* ATCC 17982
2. *Akkermansia muciniphila* ATCC BAA-835
3. *Alistipes putredinis* DSM 17216
4. *Anaerofustis stercorihominis* DSM 17244
5. *Anaerostipes caccae* DSM 14662
6. *Anaerotruncus colihominis* DSM 17241
7. *Pseudoflavonifractor capillosus* ATCC 29799
8. *Bacteroides cellulosilyticus* DSM 14838
9. *Bacteroides coprocola* DSM 17136
10. *Bacteroides dorei* DSM 17855
11. *Bacteroides eggerthii* DSM 20697
12. *Bacteroides finegoldii* DSM 17565
13. *Bacteroides intestinalis* DSM 17393
14. *Bacteroides ovatus* ATCC 8483
15. *Bacteroides pectinophilus* ATCC 43243
16. *Bacteroides plebeius* DSM 17135
17. *Bacteroides stercoris* ATCC 43183
18. *Bacteroides uniformis* ATCC 8492
19. *Bifidobacterium adolescentis* ATCC 15703
20. *Bifidobacterium adolescentis* L2-32
21. *Bifidobacterium angulatum* DSM 20098
22. *Bifidobacterium bifidum* DSM 20456
23. *Bifidobacterium breve* DSM 20213
24. *Bifidobacterium dentium* ATCC 27678
25. *Bifidobacterium longum* DJO10A
26. *Bifidobacterium longum* NCC2705
27. *Bifidobacterium longum* subsp. infantis str ATCC 15697
28. *Borrelia burgdorferi* CA-11.2A
29. *Butyrivibrio crossotus* DSM 2876
30. *Catenibacterium mitsuokai* DSM 15897
31. *Clostridium asparagiforme* DSM 15981
32. *Clostridium bartlettii* DSM 16795
33. *Clostridium bolteae* ATCC BAA-613
34. *Clostridium hiranonis* DSM 13275
35. *Clostridium leptum* DSM 753
36. *Clostridium methylpentosum* DSM 5476
37. *Clostridium nexile* DSM 1787
38. *Clostridium ramosum* DSM 1402
39. *Clostridium scindens* ATCC 35704
40. *Clostridium sp* L2-50
41. *Clostridium sp* M62/1
42. *Clostridium sp* SS2/1
43. *Clostridium spiroforme* DSM 1552
44. *Clostridium sporogenes* ATCC 15579
45. *Clostridium symbiosum* ATCC 14940
46. *Collinsella aerofaciens* ATCC 25986
47. *Collinsella intestinalis* DSM 13280
48. *Collinsella stercoris* DSM 13279
49. *Coprococcus comes* ATCC 27758
50. *Coprococcus eutactus* ATCC 27759
51. *Dorea formicigenerans* ATCC 27755
52. *Dorea longicatena* DSM 13814
53. *Eggerthella lenta* DSM 2243
54. *Enterobacter cancerogenus* ATCC 35316
55. *Eubacterium dolichum* DSM 3991
56. *Eubacterium hallii* DSM 3353
57. *Eubacterium siraeum* DSM 15702
58. *Eubacterium ventriosum* ATCC 27560
59. *Faecalibacterium prausnitzii* A2-165
60. *Faecalibacterium prausnitzii* M21/2
61. *Lactobacillus salivarius* UCC118
62. *Methanobrevibacter smithii* ATCC 35061
63. *Methanobrevibacter smithii* DSM 11975
64. *Methanobrevibacter smithii* DSM 2374
65. *Methanobrevibacter smithii* DSM 2375
66. *Mitsuokella multacida* DSM 20544
67. *Parabacteroides johnsonii*
68. *Parabacteroides merdae* ATCC 43184
69. *Parvimonas micra* ATCC 33270
70. *Photorhabdus luminescens* subsp. laumondii TTO1
71. *Prevotella copri* DSM 18205
72. *Providencia alcalifaciens* DSM 30120
73. *Providencia rettgeri* DSM 1131
74. *Providencia rustigianii* DSM 4541
75. *Roseburia faecis* M72/1
76. *Roseburia intestinalis* L1-82
77. *Ruminococcus gnavus* ATCC 29149
78. *Ruminococcus lactaris* ATCC 29176
79. *Ruminococcus obeum* ATCC 29174
80. *Ruminococcus torques* ATCC 27756
81. *Streptococcus infantarius* subsp. infantarius ATCC BAA-102
82. *Subdoligranulum variabile* DSM 15176
83. *Victivallis vadensis* ATCC BAA-548
