# Supplementary material for: Integrated immunoinformatics for the design of novel multi-epitope vaccine and identification of new drug targets against Stenotrophomonas maltophilia, a multidrug-resistant superbug
Source: J Genet Eng Biotechnol. 2026 Jul 28;24(3):100772. doi: 10.1016/j.jgeb.2026.100772 (PMC13449788; doi:10.1016/j.jgeb.2026.100772)
Supplement: Supplementary file 2 [file mmc2.docx]

**Table 1.** The promising epitopes from OMPs were used in the MEV

| Protein | Start | End | Epitope | Antigenicity (Score) | Allergenicity | Conservancy |
| --- | --- | --- | --- | --- | --- | --- |
| WP_005407892.1 | 124 | 131 | SAVAGATA | Ag (1.1272) | non-allergen | 100% |
|  | 257 | 262 | STGNDL | Ag (1.3720) | non-allergen | 100% |
|  | 254 | 261 | ADQDSSNM | Ag (1.1602) | non-allergen | 97.37% |
| WP_049451370.1 | 72 | 89 | AARAPAPALHGASTGEAT | Ag (1.1028) | non-allergen | 90% |
|  | 258 | 263 | SGKAEQ | Ag (2.6764) | non-allergen | 100% |
|  | 273 | 280 | GEESKTPS | Ag (1.9060) | non-allergen | **98.75%** |
| WP_108270537.1 | 100 | 111 | GVTSTQSDSERT | Ag (1.7579) | non-allergen | **93.83%** |
|  | 245 | 253 | DLTPDTQLS | Ag (1.1135) | non-allergen | **96.30%** |
|  | 707 | 713 | GGEARDI | Ag (1.7814) | non-allergen | **90.91%** |
| WP_169448945.1 | 986 | 993 | ELGGDRNE | Ag (1.2609) | non-allergen | 96.10% |

**Table 2.** Different arrangements of epitopes in the designed multi-epitope construct

| **No** | **Arrangement** | **Antigenicity Score** |
| --- | --- | --- |
| 1 | ADQDSSNMGPGPGSGKAEQGPGPGGEESKTPSGPGPGGVTSTQSDSERTGPGPGELGGDRNE | 1.7398 |
| 2 | ADQDSSNMGPGPGSGKAEQGPGPGGEESKTPSGPGPGELGGDRNEGPGPGGVTSTQSDSERT | 1.6926 |
| 3 | ADQDSSNMGPGPGSGKAEQGPGPGGVTSTQSDSERTGPGPGGEESKTPSGPGPGELGGDRNE | 1.7398 |
| 4 | ADQDSSNMGPGPGSGKAEQGPGPGGVTSTQSDSERTGPGPGELGGDRNEGPGPGGEESKTPS | 1.7499 |
| 5 | ADQDSSNMGPGPGSGKAEQGPGPGELGGDRNEGPGPGGEESKTPSGPGPGGVTSTQSDSERT | 1.6926 |
| 6 | ADQDSSNMGPGPGSGKAEQGPGPGELGGDRNEGPGPGGVTSTQSDSERTGPGPGGEESKTPS | 1.7499 |
| 7 | ADQDSSNMGPGPGGEESKTPSGPGPGSGKAEQGPGPGGVTSTQSDSERTGPGPGELGGDRNE | 1.7398 |
| 8 | ADQDSSNMGPGPGGEESKTPSGPGPGSGKAEQGPGPGELGGDRNEGPGPGGVTSTQSDSERT | 1.6926 |
| 9 | ADQDSSNMGPGPGGEESKTPSGPGPGGVTSTQSDSERTGPGPGSGKAEQGPGPGELGGDRNE | 1.7398 |
| 10 | ADQDSSNMGPGPGGEESKTPSGPGPGGVTSTQSDSERTGPGPGELGGDRNEGPGPGSGKAEQ | 1.7047 |
| 11 | ADQDSSNMGPGPGGEESKTPSGPGPGELGGDRNEGPGPGSGKAEQGPGPGGVTSTQSDSERT | 1.6926 |
| 12 | ADQDSSNMGPGPGGEESKTPSGPGPGELGGDRNEGPGPGGVTSTQSDSERTGPGPGSGKAEQ | 1.7047 |
| 13 | ADQDSSNMGPGPGGVTSTQSDSERTGPGPGSGKAEQGPGPGGEESKTPSGPGPGELGGDRNE | 1.7398 |
| 14 | ADQDSSNMGPGPGGVTSTQSDSERTGPGPGSGKAEQGPGPGELGGDRNEGPGPGGEESKTPS | 1.7499 |
| 15 | ADQDSSNMGPGPGGVTSTQSDSERTGPGPGGEESKTPSGPGPGSGKAEQGPGPGELGGDRNE | 1.7398 |
| 16 | ADQDSSNMGPGPGGVTSTQSDSERTGPGPGGEESKTPSGPGPGELGGDRNEGPGPGSGKAEQ | 1.7047 |
| 17 | ADQDSSNMGPGPGGVTSTQSDSERTGPGPGELGGDRNEGPGPGSGKAEQGPGPGGEESKTPS | 1.7499 |
| 18 | ADQDSSNMGPGPGGVTSTQSDSERTGPGPGELGGDRNEGPGPGGEESKTPSGPGPGSGKAEQ | 1.7047 |
| 19 | ADQDSSNMGPGPGELGGDRNEGPGPGSGKAEQGPGPGGEESKTPSGPGPGGVTSTQSDSERT | 1.6926 |
| 20 | ADQDSSNMGPGPGELGGDRNEGPGPGSGKAEQGPGPGGVTSTQSDSERTGPGPGGEESKTPS | 1.7499 |
| 21 | ADQDSSNMGPGPGELGGDRNEGPGPGGEESKTPSGPGPGSGKAEQGPGPGGVTSTQSDSERT | 1.6926 |
| 22 | ADQDSSNMGPGPGELGGDRNEGPGPGGEESKTPSGPGPGGVTSTQSDSERTGPGPGSGKAEQ | 1.7047 |
| 23 | ADQDSSNMGPGPGELGGDRNEGPGPGGVTSTQSDSERTGPGPGSGKAEQGPGPGGEESKTPS | 1.7499 |
| 24 | ADQDSSNMGPGPGELGGDRNEGPGPGGVTSTQSDSERTGPGPGGEESKTPSGPGPGSGKAEQ | 1.7047 |
| 25 | SGKAEQGPGPGADQDSSNMGPGPGGEESKTPSGPGPGGVTSTQSDSERTGPGPGELGGDRNE | 1.7433 |
| 26 | SGKAEQGPGPGADQDSSNMGPGPGGEESKTPSGPGPGELGGDRNEGPGPGGVTSTQSDSERT | 1.6961 |
| 27 | SGKAEQGPGPGADQDSSNMGPGPGGVTSTQSDSERTGPGPGGEESKTPSGPGPGELGGDRNE | 1.7433 |
| 28 | SGKAEQGPGPGADQDSSNMGPGPGGVTSTQSDSERTGPGPGELGGDRNEGPGPGGEESKTPS | 1.7534 |
| 29 | SGKAEQGPGPGADQDSSNMGPGPGELGGDRNEGPGPGGEESKTPSGPGPGGVTSTQSDSERT | 1.6961 |
| 30 | SGKAEQGPGPGADQDSSNMGPGPGELGGDRNEGPGPGGVTSTQSDSERTGPGPGGEESKTPS | 1.7534 |
| 31 | SGKAEQGPGPGGEESKTPSGPGPGADQDSSNMGPGPGGVTSTQSDSERTGPGPGELGGDRNE | 1.7433 |
| 32 | SGKAEQGPGPGGEESKTPSGPGPGADQDSSNMGPGPGELGGDRNEGPGPGGVTSTQSDSERT | 1.6961 |
| 33 | SGKAEQGPGPGGEESKTPSGPGPGGVTSTQSDSERTGPGPGADQDSSNMGPGPGELGGDRNE | 1.7433 |
| 34 | SGKAEQGPGPGGEESKTPSGPGPGGVTSTQSDSERTGPGPGELGGDRNEGPGPGADQDSSNM | 1.7310 |
| 35 | SGKAEQGPGPGGEESKTPSGPGPGELGGDRNEGPGPGADQDSSNMGPGPGGVTSTQSDSERT | 1.6961 |
| 36 | SGKAEQGPGPGGEESKTPSGPGPGELGGDRNEGPGPGGVTSTQSDSERTGPGPGADQDSSNM | 1.7310 |
| 37 | SGKAEQGPGPGGVTSTQSDSERTGPGPGADQDSSNMGPGPGGEESKTPSGPGPGELGGDRNE | 1.7433 |
| 38 | SGKAEQGPGPGGVTSTQSDSERTGPGPGADQDSSNMGPGPGELGGDRNEGPGPGGEESKTPS | 1.7534 |
| 39 | SGKAEQGPGPGGVTSTQSDSERTGPGPGGEESKTPSGPGPGADQDSSNMGPGPGELGGDRNE | 1.7433 |
| 40 | SGKAEQGPGPGGVTSTQSDSERTGPGPGGEESKTPSGPGPGELGGDRNEGPGPGADQDSSNM | 1.7310 |
| 41 | SGKAEQGPGPGGVTSTQSDSERTGPGPGELGGDRNEGPGPGADQDSSNMGPGPGGEESKTPS | 1.7534 |
| 42 | SGKAEQGPGPGGVTSTQSDSERTGPGPGELGGDRNEGPGPGGEESKTPSGPGPGADQDSSNM | 1.7310 |
| 43 | SGKAEQGPGPGELGGDRNEGPGPGADQDSSNMGPGPGGEESKTPSGPGPGGVTSTQSDSERT | 1.6961 |
| 44 | SGKAEQGPGPGELGGDRNEGPGPGADQDSSNMGPGPGGVTSTQSDSERTGPGPGGEESKTPS | 1.7534 |
| 45 | SGKAEQGPGPGELGGDRNEGPGPGGEESKTPSGPGPGADQDSSNMGPGPGGVTSTQSDSERT | 1.6961 |
| 46 | SGKAEQGPGPGELGGDRNEGPGPGGEESKTPSGPGPGGVTSTQSDSERTGPGPGADQDSSNM | 1.7310 |
| 47 | SGKAEQGPGPGELGGDRNEGPGPGGVTSTQSDSERTGPGPGADQDSSNMGPGPGGEESKTPS | 1.7534 |
| 48 | SGKAEQGPGPGELGGDRNEGPGPGGVTSTQSDSERTGPGPGGEESKTPSGPGPGADQDSSNM | 1.7310 |
| 49 | GEESKTPSGPGPGADQDSSNMGPGPGSGKAEQGPGPGGVTSTQSDSERTGPGPGELGGDRNE | 1.7264 |
| 50 | GEESKTPSGPGPGADQDSSNMGPGPGSGKAEQGPGPGELGGDRNEGPGPGGVTSTQSDSERT | 1.6792 |
| 51 | GEESKTPSGPGPGADQDSSNMGPGPGGVTSTQSDSERTGPGPGSGKAEQGPGPGELGGDRNE | 1.7264 |
| 52 | GEESKTPSGPGPGADQDSSNMGPGPGGVTSTQSDSERTGPGPGELGGDRNEGPGPGSGKAEQ | 1.6912 |
| 53 | GEESKTPSGPGPGADQDSSNMGPGPGELGGDRNEGPGPGSGKAEQGPGPGGVTSTQSDSERT | 1.6792 |
| 54 | GEESKTPSGPGPGADQDSSNMGPGPGELGGDRNEGPGPGGVTSTQSDSERTGPGPGSGKAEQ | 1.6912 |
| 55 | GEESKTPSGPGPGSGKAEQGPGPGADQDSSNMGPGPGGVTSTQSDSERTGPGPGELGGDRNE | 1.7264 |
| 56 | GEESKTPSGPGPGSGKAEQGPGPGADQDSSNMGPGPGELGGDRNEGPGPGGVTSTQSDSERT | 1.6792 |
| 57 | GEESKTPSGPGPGSGKAEQGPGPGGVTSTQSDSERTGPGPGADQDSSNMGPGPGELGGDRNE | 1.7264 |
| 58 | GEESKTPSGPGPGSGKAEQGPGPGGVTSTQSDSERTGPGPGELGGDRNEGPGPGADQDSSNM | 1.7141 |
| 59 | GEESKTPSGPGPGSGKAEQGPGPGELGGDRNEGPGPGADQDSSNMGPGPGGVTSTQSDSERT | 1.6792 |
| 60 | GEESKTPSGPGPGSGKAEQGPGPGELGGDRNEGPGPGGVTSTQSDSERTGPGPGADQDSSNM | 1.7141 |
| 61 | GEESKTPSGPGPGGVTSTQSDSERTGPGPGADQDSSNMGPGPGSGKAEQGPGPGELGGDRNE | 1.7264 |
| 62 | GEESKTPSGPGPGGVTSTQSDSERTGPGPGADQDSSNMGPGPGELGGDRNEGPGPGSGKAEQ | 1.6912 |
| 63 | GEESKTPSGPGPGGVTSTQSDSERTGPGPGSGKAEQGPGPGADQDSSNMGPGPGELGGDRNE | 1.7264 |
| 64 | GEESKTPSGPGPGGVTSTQSDSERTGPGPGSGKAEQGPGPGELGGDRNEGPGPGADQDSSNM | 1.7141 |
| 65 | GEESKTPSGPGPGGVTSTQSDSERTGPGPGELGGDRNEGPGPGADQDSSNMGPGPGSGKAEQ | 1.6912 |
| 66 | GEESKTPSGPGPGGVTSTQSDSERTGPGPGELGGDRNEGPGPGSGKAEQGPGPGADQDSSNM | 1.7141 |
| 67 | GEESKTPSGPGPGELGGDRNEGPGPGADQDSSNMGPGPGSGKAEQGPGPGGVTSTQSDSERT | 1.6792 |
| 68 | GEESKTPSGPGPGELGGDRNEGPGPGADQDSSNMGPGPGGVTSTQSDSERTGPGPGSGKAEQ | 1.6912 |
| 69 | GEESKTPSGPGPGELGGDRNEGPGPGSGKAEQGPGPGADQDSSNMGPGPGGVTSTQSDSERT | 1.6792 |
| 70 | GEESKTPSGPGPGELGGDRNEGPGPGSGKAEQGPGPGGVTSTQSDSERTGPGPGADQDSSNM | 1.7141 |
| 71 | GEESKTPSGPGPGELGGDRNEGPGPGGVTSTQSDSERTGPGPGADQDSSNMGPGPGSGKAEQ | 1.6912 |
| 72 | GEESKTPSGPGPGELGGDRNEGPGPGGVTSTQSDSERTGPGPGSGKAEQGPGPGADQDSSNM | 1.7141 |
| 73 | GVTSTQSDSERTGPGPGADQDSSNMGPGPGSGKAEQGPGPGGEESKTPSGPGPGELGGDRNE | 1.7671 |
| 74 | GVTSTQSDSERTGPGPGADQDSSNMGPGPGSGKAEQGPGPGELGGDRNEGPGPGGEESKTPS | 1.7772 |
| 75 | GVTSTQSDSERTGPGPGADQDSSNMGPGPGGEESKTPSGPGPGSGKAEQGPGPGELGGDRNE | 1.7671 |
| 76 | GVTSTQSDSERTGPGPGADQDSSNMGPGPGGEESKTPSGPGPGELGGDRNEGPGPGSGKAEQ | 1.7319 |
| 77 | GVTSTQSDSERTGPGPGADQDSSNMGPGPGELGGDRNEGPGPGSGKAEQGPGPGGEESKTPS | 1.7772 |
| 78 | GVTSTQSDSERTGPGPGADQDSSNMGPGPGELGGDRNEGPGPGGEESKTPSGPGPGSGKAEQ | 1.7319 |
| 79 | GVTSTQSDSERTGPGPGSGKAEQGPGPGADQDSSNMGPGPGGEESKTPSGPGPGELGGDRNE | 1.7671 |
| 80 | GVTSTQSDSERTGPGPGSGKAEQGPGPGADQDSSNMGPGPGELGGDRNEGPGPGGEESKTPS | 1.7772 |
| 81 | GVTSTQSDSERTGPGPGSGKAEQGPGPGGEESKTPSGPGPGADQDSSNMGPGPGELGGDRNE | 1.7671 |
| 82 | GVTSTQSDSERTGPGPGSGKAEQGPGPGGEESKTPSGPGPGELGGDRNEGPGPGADQDSSNM | 1.7548 |
| 83 | GVTSTQSDSERTGPGPGSGKAEQGPGPGELGGDRNEGPGPGADQDSSNMGPGPGGEESKTPS | 1.7772 |
| 84 | GVTSTQSDSERTGPGPGSGKAEQGPGPGELGGDRNEGPGPGGEESKTPSGPGPGADQDSSNM | 1.7548 |
| 85 | GVTSTQSDSERTGPGPGGEESKTPSGPGPGADQDSSNMGPGPGSGKAEQGPGPGELGGDRNE | 1.7671 |
| 86 | GVTSTQSDSERTGPGPGGEESKTPSGPGPGADQDSSNMGPGPGELGGDRNEGPGPGSGKAEQ | 1.7319 |
| 87 | GVTSTQSDSERTGPGPGGEESKTPSGPGPGSGKAEQGPGPGADQDSSNMGPGPGELGGDRNE | 1.7671 |
| 88 | GVTSTQSDSERTGPGPGGEESKTPSGPGPGSGKAEQGPGPGELGGDRNEGPGPGADQDSSNM | 1.7548 |
| 89 | GVTSTQSDSERTGPGPGGEESKTPSGPGPGELGGDRNEGPGPGADQDSSNMGPGPGSGKAEQ | 1.7319 |
| 90 | GVTSTQSDSERTGPGPGGEESKTPSGPGPGELGGDRNEGPGPGSGKAEQGPGPGADQDSSNM | 1.7548 |
| 91 | GVTSTQSDSERTGPGPGELGGDRNEGPGPGADQDSSNMGPGPGSGKAEQGPGPGGEESKTPS | 1.7772 |
| 92 | GVTSTQSDSERTGPGPGELGGDRNEGPGPGADQDSSNMGPGPGGEESKTPSGPGPGSGKAEQ | 1.7319 |
| 93 | GVTSTQSDSERTGPGPGELGGDRNEGPGPGSGKAEQGPGPGADQDSSNMGPGPGGEESKTPS | 1.7772 |
| 94 | GVTSTQSDSERTGPGPGELGGDRNEGPGPGSGKAEQGPGPGGEESKTPSGPGPGADQDSSNM | 1.7548 |
| 95 | GVTSTQSDSERTGPGPGELGGDRNEGPGPGGEESKTPSGPGPGADQDSSNMGPGPGSGKAEQ | 1.7319 |
| 96 | GVTSTQSDSERTGPGPGELGGDRNEGPGPGGEESKTPSGPGPGSGKAEQGPGPGADQDSSNM | 1.7548 |
| 97 | ELGGDRNEGPGPGADQDSSNMGPGPGSGKAEQGPGPGGEESKTPSGPGPGGVTSTQSDSERT | 1.8239 |
| 98 | ELGGDRNEGPGPGADQDSSNMGPGPGSGKAEQGPGPGGVTSTQSDSERTGPGPGGEESKTPS | 1.8811 |
| 99 | ELGGDRNEGPGPGADQDSSNMGPGPGGEESKTPSGPGPGSGKAEQGPGPGGVTSTQSDSERT | 1.8239 |
| 100 | ELGGDRNEGPGPGADQDSSNMGPGPGGEESKTPSGPGPGGVTSTQSDSERTGPGPGSGKAEQ | 1.8359 |
| 101 | ELGGDRNEGPGPGADQDSSNMGPGPGGVTSTQSDSERTGPGPGSGKAEQGPGPGGEESKTPS | 1.8811 |
| 102 | ELGGDRNEGPGPGADQDSSNMGPGPGGVTSTQSDSERTGPGPGGEESKTPSGPGPGSGKAEQ | 1.8359 |
| 103 | ELGGDRNEGPGPGSGKAEQGPGPGADQDSSNMGPGPGGEESKTPSGPGPGGVTSTQSDSERT | 1.8239 |
| 104 | ELGGDRNEGPGPGSGKAEQGPGPGADQDSSNMGPGPGGVTSTQSDSERTGPGPGGEESKTPS | 1.8811 |
| 105 | ELGGDRNEGPGPGSGKAEQGPGPGGEESKTPSGPGPGADQDSSNMGPGPGGVTSTQSDSERT | 1.8239 |
| 106 | ELGGDRNEGPGPGSGKAEQGPGPGGEESKTPSGPGPGGVTSTQSDSERTGPGPGADQDSSNM | 1.8588 |
| 107 | ELGGDRNEGPGPGSGKAEQGPGPGGVTSTQSDSERTGPGPGADQDSSNMGPGPGGEESKTPS | 1.8811 |
| 108 | ELGGDRNEGPGPGSGKAEQGPGPGGVTSTQSDSERTGPGPGGEESKTPSGPGPGADQDSSNM | 1.8588 |
| 109 | ELGGDRNEGPGPGGEESKTPSGPGPGADQDSSNMGPGPGSGKAEQGPGPGGVTSTQSDSERT | 1.8239 |
| 110 | ELGGDRNEGPGPGGEESKTPSGPGPGADQDSSNMGPGPGGVTSTQSDSERTGPGPGSGKAEQ | 1.8359 |
| 111 | ELGGDRNEGPGPGGEESKTPSGPGPGSGKAEQGPGPGADQDSSNMGPGPGGVTSTQSDSERT | 1.8239 |
| 112 | ELGGDRNEGPGPGGEESKTPSGPGPGSGKAEQGPGPGGVTSTQSDSERTGPGPGADQDSSNM | 1.8588 |
| 113 | ELGGDRNEGPGPGGEESKTPSGPGPGGVTSTQSDSERTGPGPGADQDSSNMGPGPGSGKAEQ | 1.8359 |
| 114 | ELGGDRNEGPGPGGEESKTPSGPGPGGVTSTQSDSERTGPGPGSGKAEQGPGPGADQDSSNM | 1.8588 |
| 115 | ELGGDRNEGPGPGGVTSTQSDSERTGPGPGADQDSSNMGPGPGSGKAEQGPGPGGEESKTPS | 1.8811 |
| 116 | ELGGDRNEGPGPGGVTSTQSDSERTGPGPGADQDSSNMGPGPGGEESKTPSGPGPGSGKAEQ | 1.8359 |
| 117 | ELGGDRNEGPGPGGVTSTQSDSERTGPGPGSGKAEQGPGPGADQDSSNMGPGPGGEESKTPS | 1.8811 |
| 118 | ELGGDRNEGPGPGGVTSTQSDSERTGPGPGSGKAEQGPGPGGEESKTPSGPGPGADQDSSNM | 1.8588 |
| 119 | ELGGDRNEGPGPGGVTSTQSDSERTGPGPGGEESKTPSGPGPGADQDSSNMGPGPGSGKAEQ | 1.8359 |
| 120 | ELGGDRNEGPGPGGVTSTQSDSERTGPGPGGEESKTPSGPGPGSGKAEQGPGPGADQDSSNM | 1.8588 |

Arrangements with highest antigenicity:

| **No** | **Arrangement** | **Score** |
| --- | --- | --- |
| 98 | ELGGDRNEGPGPGADQDSSNMGPGPGSGKAEQGPGPGGVTSTQSDSERTGPGPGGEESKTPS | 1.8811 |
| 101 | ELGGDRNEGPGPGADQDSSNMGPGPGGVTSTQSDSERTGPGPGSGKAEQGPGPGGEESKTPS | 1.8811 |
| 104 | ELGGDRNEGPGPGSGKAEQGPGPGADQDSSNMGPGPGGVTSTQSDSERTGPGPGGEESKTPS | 1.8811 |
| 107 | ELGGDRNEGPGPGSGKAEQGPGPGGVTSTQSDSERTGPGPGADQDSSNMGPGPGGEESKTPS | 1.8811 |
| 115 | ELGGDRNEGPGPGGVTSTQSDSERTGPGPGADQDSSNMGPGPGSGKAEQGPGPGGEESKTPS | 1.8811 |
| 117 | ELGGDRNEGPGPGGVTSTQSDSERTGPGPGSGKAEQGPGPGADQDSSNMGPGPGGEESKTPS | 1.8811 |

- The arrangement No. 98 was selected for designing MEV.

- **TLR4 agonist adjuvants:**

MAKLSTDELLDAFKEMTLLELSDFVKKFEETFEVTAAAPVAVAAAGAAPAGAAVEAAEEQSEFDVILEAAGDKKIGVIKVVREIVSGLGLKEAKDLVDGAPKPLLEKVAKEAADEAKAKLEAAGATVTVK

>MEV

MAKLSTDELLDAFKEMTLLELSDFVKKFEETFEVTAAAPVAVAAAGAAPAGAAVEAAEEQSEFDVILEAAGDKKIGVIKVVREIVSGLGLKEAKDLVDGAPKPLLEKVAKEAADEAKAKLEAAGATVTVKEAAAKELGGDRNEGPGPGADQDSSNMGPGPGSGKAEQGPGPGGVTSTQSDSERTGPGPGGEESKTPS

Key features of the designed multi-epitope construct. Colors indicate functional components: green represents the TLR4 agonist adjuvant, EAAAK serves as a rigid linker, GPGPG as a flexible linker, and individual epitopes (ELGGDRNE, ADQDSSNM, SGKAEQ, GVTSTQSDSERT, GEESKTPS) are indicated in their respective positions.
